# Single-dose injectable nanovaccine-in-hydrogel for robust immunotherapy of large tumors with abscopal effect

**DOI:** 10.1126/sciadv.ade6257

**Published:** 2023-07-14

**Authors:** Furong Cheng, Ting Su, Shurong Zhou, Xiang Liu, Suling Yang, Shuibin Lin, Weisheng Guo, Guizhi Zhu

**Affiliations:** ^1^ Translational Medicine Center, The Second Affiliated Hospital, Guangzhou Medical University, Guangzhou 510260, China.; ^2^ Department of Pharmaceutics and Center for Pharmaceutical Engineering and Sciences, Institute for Structural Biology and Drug Discovery, School of Pharmacy, The Developmental Therapeutics Program, Massey Cancer Center, Virginia Commonwealth University, Richmond, VA 23298, USA.; ^3^ Center for Translational Medicine, Precision Medicine Institute, The First Affiliated Hospital, Sun Yat-sen University, Guangzhou 510080, China.; ^4^ Department of Pharmaceutical Sciences, College of Pharmacy, Biointerfaces Institute, University of Michigan, Ann Arbor, MI 48109, USA.

## Abstract

Current cancer immunotherapy [e.g., immune checkpoint blockade (ICB)] only benefits small subsets of patients, largely due to immunosuppressive tumor microenvironment (TME). In situ tumor vaccination can reduce TME immunosuppression and thereby improve cancer immunotherapy. Here, we present single-dose injectable (nanovaccines + ICBs)-in-hydrogel (NvIH) for robust immunotherapy of large tumors with abscopal effect. NvIH is thermo-responsive hydrogel co-encapsulated with ICB antibodies and novel polymeric nanoparticles loaded with three immunostimulatory agonists for Toll-like receptors 7/8/9 (TLR7/8/9) and stimulator of interferon genes (STING). Upon in situ tumor vaccination, NvIH undergoes rapid sol-to-gel transformation, prolongs tumor retention, sustains the release of immunotherapeutics, and reduces acute systemic inflammation. In multiple poorly immunogenic tumor models, single-dose NvIH reduces multitier TME immunosuppression, elicits potent TME and systemic innate and adaptive antitumor immunity with memory, and regresses both local (vaccinated) and distant large tumors with abscopal effect, including distant orthotopic glioblastoma. Overall, NvIH holds great potential for tumor immunotherapy.

## Teaser

Nanoparticle-in-hydrogel composites were developed to co-deliver immunotherapeutic cocktails to treat large tumor, near and far.

## INTRODUCTION

Cancer immunotherapy has become a major therapy modality for many types of cancers (*1*, *2*). However, current immunotherapy, such as immune checkpoint blockade (ICB), has only benefited a small fraction of patients (*3*, *4*), in part due to the immunosuppressive tumor microenvironment (TME) and the resulting insufficient tumor infiltration of antitumor T cells (*5*–*7*). Various approaches have been studied to address these challenges, ranging from cancer vaccines (*8*, *9*), chemotherapy (*10*, *11*), radiotherapy (*12*), to oncolytic virotherapy (*13*, *14*). Although cancer therapeutic vaccines based on exogenous cancer antigens [e.g., tumor-associated antigens (TAAs) and neoantigens] are attractive to elicit antigen-specific antitumor T cell responses (*15*, *16*), broad clinical adoption of this approach is likely hampered by the high inter- and intrapatient tumor antigenic heterogeneity, time-consuming and costly antigen identification and manufacturing, and often poor co-delivery of immunostimulant adjuvants and antigens to antigen-presenting cells (APCs) for the optimal T cell responses with memory (*17*–*23*). By intratumoral injection of immunomodulators, in situ vaccination holds the potential to reduce TME immunosuppression and elicit systemic antitumor immunity against various in situ antigens in tumor tissues (*24*–*27*). Various immunostimulants such as Toll-like receptor (TLR) agonists and stimulator of interferon genes (STING) agonists have been preclinically or clinically tested for in situ tumor vaccination (*28*–*30*). Although promising, these immunostimulants have shown suboptimal therapeutic efficacy thus far, largely due to poor tumor retention and rapid systemic dissemination, lack of effective TAA cross-presentation, and limited intracellular delivery, thus requiring high and frequent doses (*30*–*34*).

**Fig. 1.**
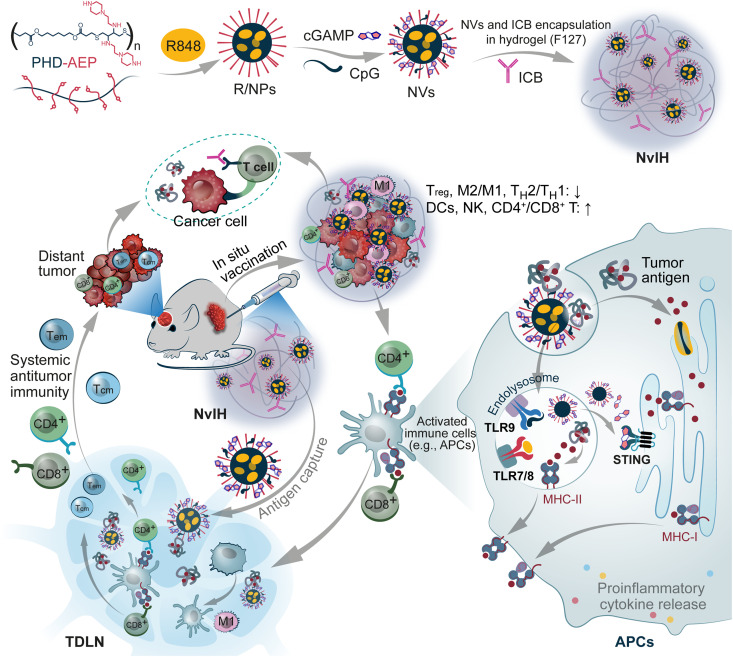
In situ vaccination with single-dose NvIH reduced TME immunosuppression, enhanced TME antitumor immune milieu, and elicited systemic antitumor immunity, resulting in robust immunotherapy of large poorly immunogenic tumors with abscopal effect. NvIH is composed of injectable (NVs + ICBs)-in-hydrogel that was loaded with triple immunostimulants (TLR7/8/9 and STING agonists R848, CpG, and cGAMP) and anti–programmed death receptor (αPD-1) antibody, and prolongs the tumor retention of these immunotherapeutics. Single-dose intratumoral injection of NvIH remodeled immune milieu, increased tumor antigen accumulation in TDLNs, and elicited potent systemic antigen-specific antitumor T cell responses with memory. Single-dose NvIH substantially inhibited and regressed large poorly immunogenic tumors with abscopal effect that inhibited distant tumors, including orthotopic glioblastoma. NVs, nanovaccines; NvIH, NvH loaded with ICB agents; TDLN, tumor-draining lymph node; APC, antigen-presenting cell; TLR, Toll-like receptor; MHC, major histocompatibility complex; T_CM_, central memory T cell; T_EM_, effector memory T cell; T_H_, helper T cell; T_reg_, regulatory T cell; NK cell, natural killer cell.

As a step to address the above challenges, here, we first developed nanovaccines (NVs) for simultaneous antigen cross-presentation and multi-pronged pattern recognition receptor (PRR) antitumor immunostimulation. Biomaterials such as macroscale hydrogel and nanocarriers have shown great potential to prolong the retention of chemical and biologic therapeutics while reducing off-target adverse side effects (*35*–*37*). Thus, we then developed NvIH, an injectable thermoresponsive (NVs + ICBs)-in-hydrogel composites that are composed of triple-immunostimulant NVs (TLR7/8/9 and STING agonists) and ICB agents, for robust and durable combination cancer immunotherapy (Fig. 1). 2',3'-cyclic guanosine monophosphate-adenosine monophosphate (cGAMP) activates STING to elicit antitumor type I interferon responses (IFN-I) (*38*). Thus, the combination of TLR7/8/9 and STING agonists is expected to activate multiple synergistic pathways (*39*); further, such combination can broaden the scope of cell subsets that can be activated, since TLR7/8/9 and STING are expressed in differential cell subsets (*40*). As a result, these triple combination immunostimulants are expected to elicit optimal antitumor immunostimulation. However, the therapeutic efficacy of these molecular immunostimulant agonists is limited in part by their poor pharmacokinetics (*30*, *41*). To address this challenge, we first synthesized a series of self-assembled polymeric nanoparticles (NPs) and identified NPs as NV carriers for efficient intracellular immunostimulant co-delivery to APCs for immunostimulation. Then, we loaded NVs and ICB agents in U.S. Food and Drug Administration–approved hydrogel Pluronic, and the resulting NvIH notably prolonged the tumor retention of these immunotherapeutics. Single-dose intralesional injection of NvIH remodeled immune milieu and reduced cellular and molecular immunosuppression in TME, increased tumor antigen draining and accumulation in tumor-draining lymph nodes (TDLNs), and elicited potent systemic antigen-specific antitumor T cell responses with memory. As a result, NvIH substantially inhibited and regressed large tumors with abscopal effect, which potentiated the immunotherapy of distant poorly immunogenic tumors, including distant orthotopic glioblastoma. These results underscore the potential of NvIH for safe and robust immunotherapy of local and distant large tumors.

## RESULTS

### Fabrication and characterization of NvH

**Fig. 2.**
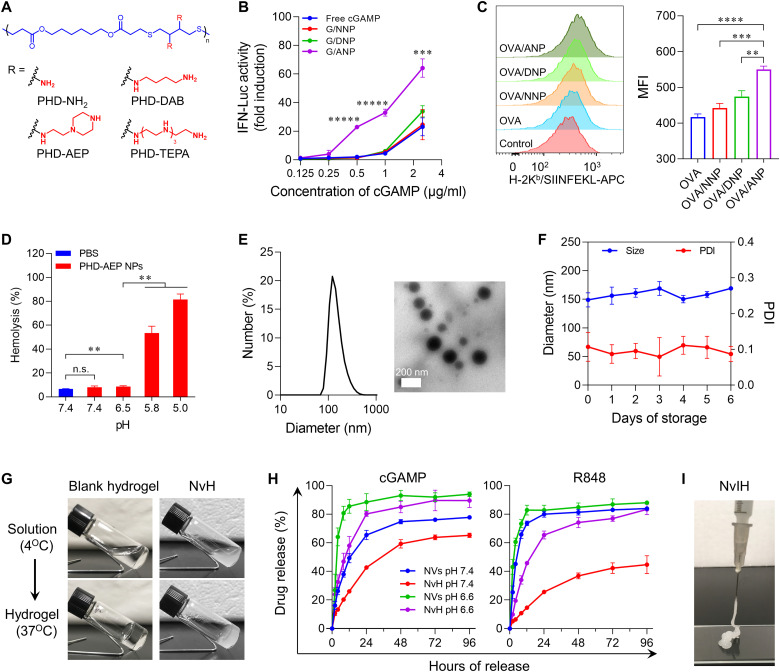
NvH synthesis and characterization. (**A**) Chemical structures of four polymers with different substituent modifications screened for triple immunostimulant co-delivery. (**B**) cGAMP-loaded NPs outperformed free cGAMP to induce IFN-I responses in RAW-ISG macrophage reporter cells (treatment: 24 hours; *n* = 4). (**C**) Representative flow cytometry histogram (left) and mean fluorescence intensity (MFI) (right) of the MHC-I/SIINFEKL complex levels on DC2.4 cells that were incubated with the indicated OVA formulations (*n* = 3). (**D**) Hemolysis assay results showing the pH-dependent membrane rupture activity of PHD-AEP NPs (30 μg/ml, 37°C, 1 hour; *n* = 3). (**E**) Hydrodynamic diameters and a TEM image of NVs. (**F**) Hydrodynamic diameters of NVs stored in water over 6 days, indicating good stability (1 mg/ml, ambient temperature; *n* = 3). (**G**) Gelation of blank hydrogel and NvH using a vial-tilting method. (**H**) In vitro release kinetics of R848 and cGAMP from NVs and NvH (*n* = 3). (**I**) Photograph of syringe injection of NvIH. ICB agent in NvIH was αPD-1. PHD-NH_2_ NPs: NNP; PHD-DAB NPs: DNP; PHD-AEP NPs: ANP. Data: mean ± SD. *P* values were determined by two-way ANOVA, Tukey’s multiple-comparison test (**P < 0.01; ***P < 0.001; ****P < 0.0001; *****P < 0.00001).

Given the typically large mesh pores in injectable Pluronic hydrogel (*42*), we hypothesize that encapsulation of immunostimulant-loaded NVs in hydrogel would mediate the controlled release and prolong the tissue retention of these immunostimulants (*35*). To synthesize immunostimulant-loaded NVs, we designed cationic polymers to form NPs with a hydrophobic core for R848 (R) loading and a brush cationic surface for the loading of CpG (C) and cGAMP (G). The charge density of cationic polymers determines nucleic acid loading efficiency and charge-induced toxicity (*43*). To develop polymeric nanocarriers with minimal toxicity and high immunostimulant loading efficiency, we synthesized a series of cationic polymers (Fig. 2A) using thiol-Michael addition polymerization and substitution reactions (figs. S1 and fig. S2) (*44*). First, poly(β-thioether esters) PHD was synthesized and confirmed by ^1^H nuclear magnetic resonance (NMR) (fig. S2A), with molecular weight (*M*_n_) of 14.7 kDa as determined by gel permeation chromatography (GPC) (fig. S2B). Subsequently, cationic groups were grafted to the backbone to prepare cationic polymers poly(β-thioether esters)-amine (PHD-NH_2_), PHD-1,4-diaminobutane (PHD-DAB), PHD-1-(2-aminoethyl) piperazine (PHD-AEP), and PHD-tetraethylenepentamine (PHD-TEPA) (Fig. 2A and fig. S1). The cationic group grafting rates of PHD-NH_2_, PHD-DAB, PHD-AEP, and PHD-TEPA were 71%, 75%, 66%, and 60%, respectively (fig. S3). These polymers were self-assembled into NPs in water (fig. S4). Meanwhile, the NPs were degradable, with nearly 50% ester bond in PHD-AEP NP hydrolysis in phosphate-buffered saline (PBS) over 10 days (fig. S5), suggesting the eventual degradation of PHD-AEP NPs in vivo. In general, the increase of electrostatic charge of these polymers aggravated their cytotoxicity, and NPs such as PHD-TEPA induced strong cytotoxicity due to high zeta potential of NPs (~52.3 mV) (fig. S6). PHD-NH_2_, PHD-DAB, and PHD-AEP NPs showed low cytotoxicity and were selected for further studies.

We then evaluated the ability of NPs for the intracellular delivery of immunostimulants. We used 2′-[DY-547]-AHC-c-diGMP (CDG^DY547^) as a model immunostimulant, which is structurally similar to cGAMP. PHD-AEP NPs significantly increased CDG^DY547^ uptake by ~3.8- to 13.5-fold, in contrast to only ~1.2- to 1.9-fold and ~1.7- to 2.9-fold of increase by PHD-NH_2_ and PHD-DAB NPs, respectively (fig. S7). Consistently, in RAW-Lucia ISG cells, cGAMP-loaded PHD-AEP NPs significantly outperformed PHD-NH_2_ and PHD-DAB NPs to enhance dose-dependent IFN-I response (Fig. 2B).

An ideal in situ tumor vaccination is expected to not only remodel immune milieu in TME but also elicit systemic antitumor T cell responses. We then evaluated the ability of NVs to capture antigens in vitro, which would allow NPs to further deliver tumor antigens to APCs for antigen presentation and antitumor CD4^+^ and/or CD8^+^ T cell responses. First, to determine whether NPs captured antigens, NPs were mixed with ovalbumin (OVA) as a model antigen, and NP-bound OVA was quantified by bicinchoninic acid (BCA) protein assay. All the three NPs, especially PHD-AEP NPs, captured OVA, which is enhanced by NP/antigen mass ratios (fig. S8). Next, DC2.4 dendritic cells (DCs) were treated with OVA-loaded NPs for 24 hours and then stained using an antibody against OVA_257–264_ (SIINFEKL) complexed with H-2K^b^ major histocompatibility complex I (MHC-I). Flow cytometric analysis of this H-2K^b^/SIINFEKL complex showed that, while relative to free OVA, all three NPs enhanced SIINFEKL presentation on DCs (Fig. 2C). OVA-loaded PHD-AEP NPs significantly outperformed OVA-loaded PHD-NH_2_ and PHD-DAB NPs. Collectively, PHD-AEP NPs showed low cytotoxicity, enhanced cGAMP activity, and efficiently captured antigens and enhanced antigen presentation. Thus, PHD-AEP NPs were selected for further studies.

We then loaded triple immunostimulants for their co-delivery and synergistic immunostimulation. To deliver cGAMP for the cytosolic activation of STING, we first demonstrated the ability of PHD-AEP NPs for pH-responsive endosome escape, as shown by their pH-responsive erythrocyte membrane destabilization (Fig. 2D) (*9*). Next, we prepared R848-loaded NPs (R/NPs) by solvent evaporation. The dynamic light scattering (DLS) and transmission electron microscopy (TEM) showed that R/NPs had uniform sizes, with a hydrodynamic diameter of ~97 nm (fig. S9A). The zeta potential of R/NPs was ~37.2 mV (fig. S9B). Moreover, R/NPs had good stability at ambient temperature (fig. S9C). Next, R/NPs were loaded with CpG and cGAMP by electrostatic interaction, as verified by a gel retardation assay (fig. S10). The diameter of the resulting NPs loaded with R848, CpG, and cGAMP (CGR/NPs or NVs) increased to ~149 nm (Fig. 2E) with good stability (Fig. 2F).

To prolong the release duration of immunostimulants, thermosensitive hydrogel Pluronic was used to prepare the NVs-in-hydrogel as NvH. Gelation study demonstrated that NV encapsulation in hydrogel did not interfere with gel formation (Fig. 2G). NvH underwent rapid solution (at 4°C) to gel (at 37°C) (sol-to-gel) transformation within 30 s, making it suitable for syringe injection. We then studied immunostimulant release kinetics from NvH. NVs had burst release in the first 4 hours, and within 12 hours, 74% and 83% R848 as well as 49% and 86% cGAMP were released at pH 7.4 and pH 6.6, respectively (Fig. 2H). Relative to NVs, NvH exhibited controlled drug release and took over fourfold longer to release equivalent amounts of drugs. Moreover, relative to drug release kinetics at physiological pH 7.4, an acidic condition (pH 6.6) facilitated drug release from NvH (Fig. 2H), indicating the potential of NvH for efficient release of immunostimulants in the slightly acidic TME relative to healthy tissues. Moreover, NvH was also loaded with an ICB agent, αPD-1, to prepare NvIH for the optimal immunotherapeutic efficacy of tumor due to the synergistic antitumor immunomodulatory effects of PRR immunostimulation and ICB (Fig. 2I). The syringe-injectable hydrogel used in NvH allows for surgery-free minimally invasive syringe injection, followed by fast gelation at physiological temperature that allows prolonged tumor retention and sustained release of drugs from the NvH reservoir.

### Robust APC activation by NVs and NvH

**Fig. 3.**
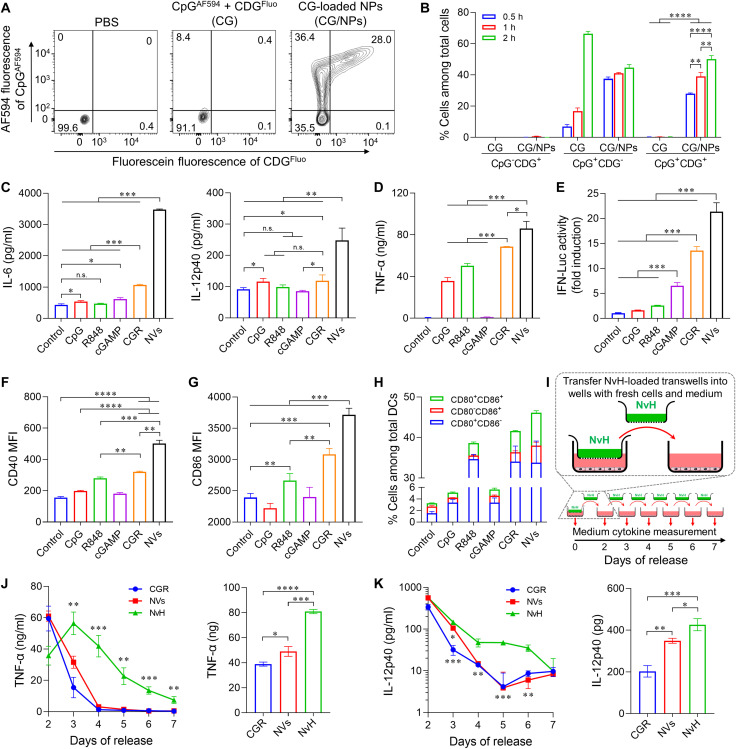
Robust in vitro immunostimulation by NvH. (**A**) Representative flow cytometry dot plots of RAW264.7 macrophages treated with PBS, CG, or CG/NPs (0.5 hour). (**B**) Percent of RAW264.7 macrophages that took up CpG and/or CDG after incubation with CG or CG/NPs for 0.5 to 2 hours (*n* = 3). (**C**) ELISA results showing IL-6 and IL-12p40 concentrations in the medium of as-treated DC2.4 cells (CpG: 50 nM; R848: 150 nM; cGAMP: 1 μg/ml; treatment: 24 hours; *n* = 3). (**D**) ELISA results showing the TNF-α concentration in as-treated RAW264.7 macrophages (CpG: 50 nM; R848: 150 nM; cGAMP: 1 μg/ml; treatment: 24 hours; *n* = 3). (**E**) IFN response in RAW-Lucia ISG macrophage reporter cells, indicating robust IFN-I responses induced by NVs (CpG: 50 nM; R848: 150 nM; cGAMP: 1 μg/ml; treatment: 24 hours; *n* = 4). (**F** and **G**) MFI of costimulatory factors CD40 (F) and CD86 (G) on as-treated DC2.4 cells, as quantified by flow cytometry (*n* = 3). (**H**) Percentage of CD80- and/or CD86-positive DC2.4 cells treated as above. Data were quantified by flow cytometry (*n* = 3). (**I**) Schematic illustration of NvH in vitro immunostimulation of RAW264.7 macrophages. (**J** and **K**) Left: ELISA results for TNF-α (J) and IL-12p40 (K) concentrations from RAW264.7 macrophages cocultured with NvH or controls in transwells for 0 to 7 days. Right: AUC of the cytokine concentrations over 7 days of treatment (*n* = 3). Data: mean ± SD. *P* values were determined by two-way ANOVA, Tukey’s multiple-comparison test (n.s., not significant; *P < 0.05; **P < 0.01; ***P < 0.001; ****P < 0.0001).

Co-delivery of immunostimulants is expected to elicit potent antitumor immunostimulation (*45*). We first evaluated the immunostimulant co-delivery efficiency by NPs. Alexa Fluor 594–labeled CpG (CpG^AF594^; C) and 2′-Fluo-AHC-c-diGMP (CDG^Fluo^; G)–loaded NPs (CG/NPs) showed efficient intracellular co-delivery, relative to physically mixed free CDG^Fluo^ + CpG^AF594^ (CG) in RAW264.7 cells (0.5 hour) (Fig. 3A and fig. S11). The co-delivery ratio (CpG^+^CDG^+^) of CG/NP-treated cells increased with time, in contrast to negligible CpG^+^CDG^+^ CG-treated cells (0.3%) even after 2 hours, as shown by flow cytometry (Fig. 3B) and verified by confocal microscopy (fig. S12).

Next, we evaluated the ability of NVs and NvH for APC activation. NV treatment of DC2.4 cells for 24 hours significantly enhanced the expression of proinflammatory factors interleukin-6 (IL-6), IL-12, tumor necrosis factor–α (TNF-α), and IFN-I, achieving 3.3-, 2.1-, 1.3-, and 1.6-fold increases, respectively, relative to mixed free CpG + cGAMP + R848 (CGR) (Fig. 3, C to E, and fig. S13). Further, NVs promoted the expression of MHC-II and costimulatory factors CD40, CD80, and CD86 on DCs (fig. S14 and Fig. 3, F and G). In addition, NVs resulted in 8.09% CD80^+^CD86^+^ DCs, a 55% increase relative to CGR (Fig. 3H and fig. S15), which indicates strong immunoactivation induced by NVs. To test our hypothesis that hydrogel prolongs the immunostimulatory efficacy of NVs, RAW264.7 macrophages were treated with free CGR, NVs, and NvH in transwells that mimic the physiological environment in TME (Fig. 3I). While NVs efficiently induced the production of only TNF-α and IL-12p40 in the first 2 days, NvH efficiently elicited prolonged TNF-α and IL-12p40 production for up to 7 days (Fig. 3, J and K). The total expression [area under curve (AUC)] of TNF-α and IL-12 treated by NvH increased by 1.7- and 1.4-fold compared with that of NVs, respectively. Overall, NVs enhanced proinflammatory responses and DC maturation, which, together with NVs’ ability to capture tumor antigens, allow NVs to elicit robust antitumor T cell responses.

### Prolonged tumor retention of immunostimulants and potent antitumor immunity by in situ tumor vaccination with single-dose NvH

**Fig. 4.**
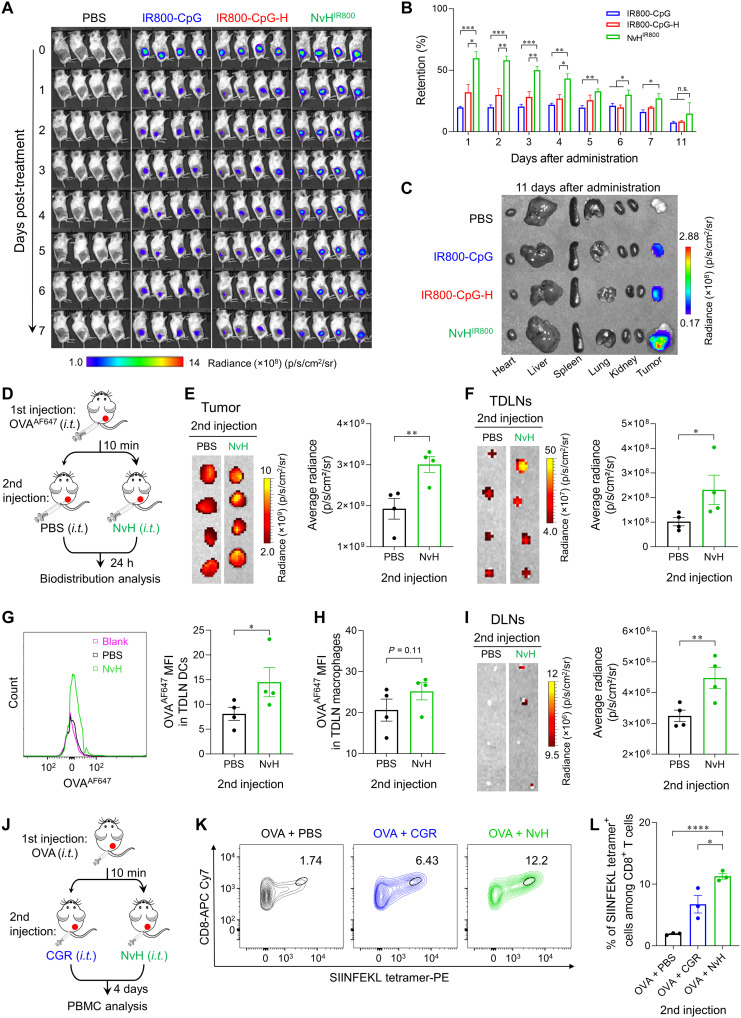
In situ tumor vaccination with single-dose NvH for prolonged tumor retention of immunostimulants for potent antitumor immunity. (**A**) IVIS images of 4T1 tumor–bearing Balb/c mice over 1 to 7 days after intratumoral (i.t.) administration of CpG-IR800–loaded NvH (NvH^IR800^) and controls (*n* = 4). (**B**) Quantification of IR800-CpG fluorescence intensities in the above 4T1 tumors. Data were normalized to the tumor fluorescence intensities measured immediately after injection. (**C**) IVIS images of ex vivo major organs and tumors collected 11 days after administration. (**D**) Experimental design of antigen capturing and delivery by NvH. (**E** and **F**) IVIS images (left) and quantification of OVA^AF647^ average fluorescence intensities (right) in ex vivo tumor I and TDLNs (F). (**G**) Representative flow cytometry histogram (left) and quantification (right) of OVA^AF647^ uptake by TDLN DCs. (**H**) Quantification of OVA^AF647^ uptake by TDLN macrophages. (**I**) IVIS images (left) and quantification of OVA^AF647^ average fluorescence intensities (right) in ex vivo DLNs. (**J**) Experimental design of antigen-specific T cell response elicited by in situ tumor vaccination with NvH. (**K** and **L**) Representative flow cytometry plots (K) and quantification (L) of the H-2K^b^-SIINFEKL tetramer staining results showing that NvH augments peripheral SIINFEKL-specific CD8^+^ T cells in mice (day 4). Data: mean ± SEM. *P* values were determined by one-way (B) and (E) to (I) or two-way (L) ANOVA, Tukey’s multiple-comparison test (*P < 0.05; **P < 0.01; ***P < 0.001; ****P < 0.0001).

Long tumor retention of immunostimulants is expected to enhance their antitumor immunomodulatory efficacy while reducing their systemic toxicity caused by systemically disseminated immunostimulants (*37*). First, 6 days after subcutaneous injection of NP-H and PBS control in Balb/c mice, NP-H did not cause significant changes of mouse body weight, major blood immune cell counts, serum TNF-α, or major organ anatomy (fig. S16). The results indicated the overall good safety of NP-H. We then evaluated the ability of NvH for tumor retention and distribution of immunostimulants. One to 7 days after intratumoral administration, as shown by IVIS imaging of mice, IR800-CpG loaded in NvH (NvH^IR800^) enhanced tumor retention of IR800-CpG relative to free IR800-CpG and IR800-CpG loaded in hydrogel alone (IR800-CpG-H) (Fig. 4, A and B). For instance, 1 day after administration, NvH^IR800^ showed 3.1- and 1.9-fold enhanced tumor retention relative to free IR800-CpG and IR800-CpG-H, respectively (Fig. 4B). Eleven days after administration, ex vivo tissue imaging shows that NvH^IR800^ still retained a significant amount of CpG in the tumor without detectable CpG accumulation in any healthy tissues, indicating the potential of in situ vaccination with NvH to reduce adverse side effects caused by randomly disseminated immunostimulants (Fig. 4C). Near-infrared dye DIR and CDG^DY547^ were used as fluorescence probes to mimic R848 and cGAMP, respectively. The IVIS imaging and quantification of fluorescence intensities showed that DIR-loaded NvH (NvH^DIR^) sustained DIR release and prolonged DIR tumor retention for at least a week after a single injection, while free DIR was rapidly cleared (fig. S17, A and B). Likewise, NvH also prolonged the tumor retention of CDG^DY547^, with a 2.8-fold tumor DY547 fluorescence intensity relative to free CDG^DY547^ on day 5 (fig. S17, C and D).

Encouraged by the ability of NVs to capture antigens in vitro, we then studied whether NvH captured tumor antigens and deliver them to APCs in vivo to activate antitumor antigen-specific T cell responses. With AF647-labeled OVA (OVA^AF647^) as a model antigen, 4T1 tumor–bearing Balb/c mice were intratumorally injected with free OVA^AF647^, followed by intratumoral injection of PBS or NvH 10 min later. After 24 hours, the distribution of OVA^AF647^ in tumors and inguinal lymph nodes was measured by IVIS imaging and flow cytometry (Fig. 4D). NvH efficiently captured OVA^AF647^ in situ (Fig. 4E) and enhanced OVA^AF647^ delivery to TDLNs (Fig. 4F) and distal inguinal lymph nodes (DLNs) (Fig. 4I), with 2.3- and 1.4-fold higher OVA accumulation in TDLNs and DLNs by NvH relative to background (Fig. 4, F and I). Further, flow cytometry showed that OVA^AF647^ was efficiently taken up by TDLN APCs (e.g., DCs and macrophages) (Fig. 4, G and H). To further evaluate whether in situ NvH vaccination of tumor activates antigen-specific T cells, 4T1 tumor–bearing Balb/c mice were intratumorally injected with free OVA, and 10 min later, CGR or NvH was intratumorally injected, respectively (Fig. 4J). Four days later, H-2K^b^-SIINFEKL tetramer staining of peripheral blood mononuclear cells (PBMCs) showed systemic SIINFEKL-specific CD8^+^ T cell responses induced by NvH (Fig. 4K), a 1.7-fold increase relative to that induced by free CGR (Fig. 4L). Further, 4 days after administration, intratumoral NvH increased cytokine^+^CD8^+^ T cells by 3.9- and 3.1-fold relative to PBS and CGR, respectively (fig. S18).

**Fig. 5.**
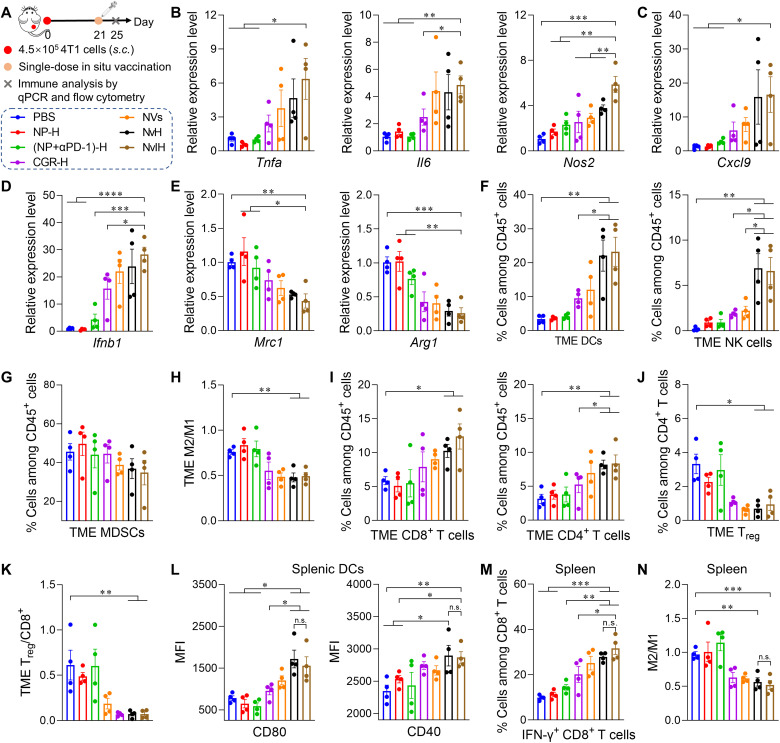
In situ vaccination with single-dose NvH and NvIH enhanced antitumor immune responses and reduced immunosuppression in TME and systemically. (**A**) Experimental design. Balb/c mice were inoculated subcutaneously (s.c.) with 4.5 × 10^5^ 4T1 cells on the right flank. On day 21, mice were treated intratumorally with (NVs + αPD-1)-loaded in hydrogel (NvIH) or controls, and tumors and spleens were isolated for immune analysis on day 25. (**B** to **E**) qPCR results for cytokines and chemokines in 4T1 tumors indicate that NvH and NvIH enhanced the transcription of proinflammatory genes *Tnfa*, *Il6*, *Nos2, Ifnb1,* and *Cxcl9* and reduced the transcription of tumor immunosuppressive genes *Arg1* and *Mrc1*. (**F** to **K**) 4T1 tumor immune milieu analysis indicates that NvH and NvIH increased antitumor immune cells and reduced tumor immunosuppressive MDSCs, T_reg_, M2/M1-like macrophage ratios, and T_reg_/CD8^+^ T cell ratios. (**L** to **N**) NvH and NvIH enhanced CD40 and CD80 expression in splenic DCs (L), enhanced IFN-γ–expressing functional CD8^+^ T cells (M), and reduced M2/M1-like macrophage ratios (N), indicating systemic antitumor immunomodulation. Data: mean ± SEM (*n* = 4). *P* values were determined by two-way ANOVA followed by Tukey’s multiple-comparison test (n.s., not significant; *P < 0.05; **P < 0.01; ***P < 0.001; ****P < 0.0001).

TME is primarily where tumor cells suppress antitumor immune cells (*6*, *7*). We tested NvH to modulate antitumor immunity in 4T1 mammary cancer TME (Fig. 5A). Single-dose intratumoral NvH dramatically inhibited the growth of tumor (fig. S19), which was further analyzed by quantitative polymerase chain reaction (qPCR) of the transcription levels of key immune markers. As a result, intratumoral NvH enhanced the expression of proinflammatory M1-like macrophage-associated genes (*Tnfa*, *Il6*, and *Nos2*; Fig. 5B) and decreased expression of immunosuppressive M2-like macrophage-associated *Arg1* and *Mrc1* (Fig. 5E), and such immunomodulation was further enhanced by αPD-1–loaded NvH (NvIH). Moreover, NvIH increased the gene expression of proinflammatory *Ifnb1* (27.6-fold increase relative to PBS) and chemokine *Cxcl9* that promotes the chemotaxis of antitumor T cells to TME (13.8-fold increase relative to PBS) (Fig. 5, C and D). Next, we evaluated the impact of NvIH on the immune cell milieu of the 4T1 TME by flow cytometry (fig. S20). Single-dose intratumoral NvH and NvIH, respectively, increased the percentage of tumor-infiltrating DCs (by 2.3- and 2.4-fold) and natural killer (NK) cells (by 3.7- and 3.5-fold) (Fig. 5F) while reducing the percentage of myeloid-derived suppressor cells (MDSCs) (by 17.2% and 21.7% reduction) relative to CGR-H (Fig. 5G). Moreover, NvH and NvIH repolarized macrophages by reducing the M2/M1 ratio (Fig. 5H). The percentages of tumor-infiltrating CD8^+^ and CD4^+^ T cells, respectively, were increased to 10.3% and 8.2% by NvH, and 12.4% and 8.4% by NvIH (Fig. 5I). Prevalent tumor regulatory T cells (T_reg_) is associated with poor patient prognosis (*6*). Single-dose intratumoral NvIH reduced TME T_reg_ (Fig. 5J) and the TME T_reg_/CD8^+^ T cell ratio (Fig. 5K). Together, these results suggest that single-dose NvH, alone or combined with αPD-1, remodeled molecular and cellular immune milieu with enhanced antitumor immunity and reduced tumor immunosuppression in the TME.

Given that NvH captured and delivered antigens to APCs and elicited antigen-specific T cells, we investigated the systemic immune modulation by NvH by analyzing splenic immune milieu. Single-dose intratumoral NvH increased the expression of CD40 and CD80 on splenic DCs by 1.3- and 2.2-fold relative to PBS, respectively, suggesting the maturation of splenic DCs that can promote antitumor T cell responses (Fig. 5L). Further, 4 days after administration, relative to PBS, intratumoral NvH increased IFN-γ^+^CD8^+^ T cells in spleen by 2.8-fold (Fig. 5M) and decreased the splenic M2/M1 ratio by 42% (Fig. 5N). NvIH further enhanced CD8^+^ T cell activation and macrophage repolarization in the spleen (Fig. 5, M and N).

### Potent antitumor immunomodulation and immunotherapy of large tumors by in situ vaccination with single-dose NvH

**Fig. 6.**
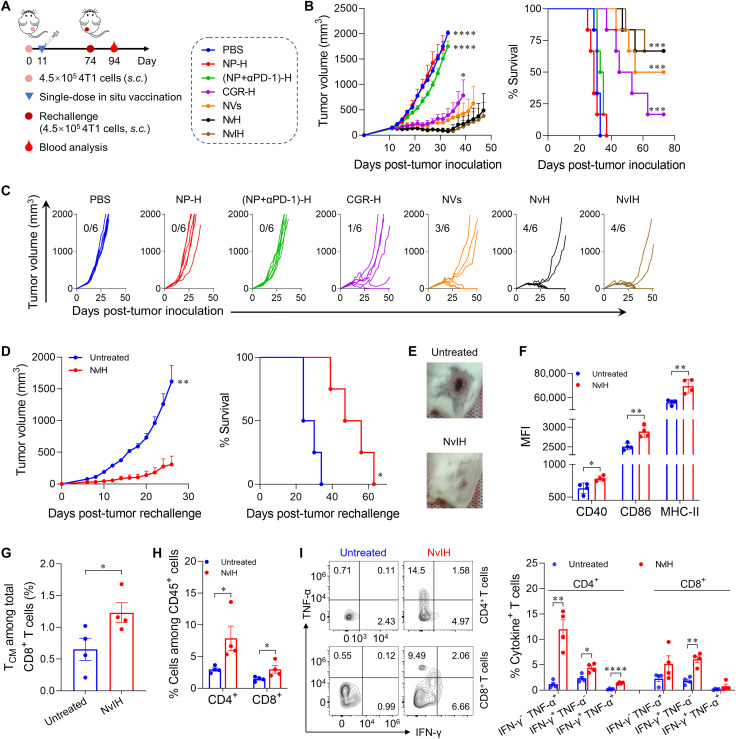
In situ tumor vaccination with single-dose NvH for efficient antitumor immunomodulation and robust immunotherapy of large tumors. (**A**) Experimental design. Balb/c mice were inoculated subcutaneously with 4.5 × 10^5^ 4T1 cells on the right flank. On day 11, mice were treated intratumorally with (NVs + αPD-1)-loaded in hydrogel (NvIH) or controls. On day 74, mice with complete tumor regression were rechallenged with 4T1 cells, and the PBMCs were collected and analyzed on day 94 (20 days after tumor rechallenge). (**B** and **C**) Tumor volumes and mouse survival curves (B), and individual tumor growth curves (C) of treated Balb/c mice with large 4T1 tumors (~150 mm^3^) (*n* = 6). Fractions: complete regression rates. (**D**) Mice with complete tumor regression were rechallenged with 4T1 cells on the contralateral flank 74 days after tumor inoculation. Mice in the untreated group were age-matched (*n* = 4). (**E**) Representative photographs of rechallenged tumors on day 94 after first tumor inoculation (20 days after tumor rechallenge). (**F** to **I**) Analysis of PBMC immune cells from rechallenged mice. (F) MFI of MHC-II and costimulatory factors CD40 and CD86 on DCs from PBMCs. (G) and (H) Fractions of PBMC T_CM_ and CD4^+^ and CD8^+^ T cells. (I) Representative flow cytometry dot plots and quantification of cytokine^+^ T cells indicate that NvIH enhanced functional CD4^+^ and CD8^+^ T cells. Data: mean ± SEM. *P* values were determined by two-way ANOVA, Tukey’s multiple-comparison test; log-rank (Mantel-Cox) test was used for animal survival comparison (*P < 0.05; **P < 0.01; ***P < 0.0001; ****P < 0.0001).

Next, we evaluated the therapeutic efficacy of NvH in Balb/c mice subcutaneously inoculated with large poorly immunogenic 4T1 tumors (~150 mm^3^) (Fig. 6A). NP-H showed negligible tumor growth inhibition [doubling time (DT)_NP-H_ = 4.4 days] relative to PBS (DT_PBS_ = 3.7 days). By contrast, single-dose in situ tumor vaccination with NvH significantly decreased the tumor growth rate (DT_NvH_ = 30.5 days) and increased mouse survival relative to NVs (DT_NVs_ = 21.2 days) and immunostimulant-loaded hydrogel CGR-H (DT_CGR-H_ = 17.0 days) (Fig. 6B). Further, while αPD-1 loaded in NP-H only slightly inhibited tumor growth [DT_(NP+αPD-1)-H_ = 5.1 days], NvIH dramatically inhibited tumor growth (DT_NvIH_ = 33.6 days) and prolonged mouse survival, with ~85% of mice surviving 60 days after tumor inoculation (Fig. 6B). Remarkably, single-dose NvH and NvIH completely regressed four of six tumors, without any tumor residuals or recurrence up to day 74 after inoculation (Fig. 6C). Next, when rechallenged with 4T1 cells on the opposite flank relative to the regressed tumor, NvIH-treated mice with regressed tumors largely resisted tumor rechallenge (Fig. 6, D and E). To study the immune status underlying the resistance to tumor rechallenge in the above mice, 20 days after rechallenge, we observed the enhanced expression of MHC-II, CD40, and CD86 on DCs from PBMCs (Fig. 6F). In addition, NvIH resulted in a 1.9-fold higher fraction of PBMC CD8^+^ central memory T cells (T_CM_) compared to the untreated mice (Fig. 6G). Last, NvIH enhanced the fraction of circulating CD4^+^ and CD8^+^ T cells and cytokine-secreting (IFN-γ, TNF-α) CD4^+^ and CD8^+^ T cells, which contributed to the protective immunity against tumor rechallenge (Fig. 6, H and I). To further study the therapeutic roles of key lymphocytes, αNK1.1, αCD4, and αCD8α antibodies were intraperitoneally administered to deplete the corresponding lymphocytes in tumor-bearing mice treated with NvIH as above. As a result, all these antibodies partially reduced the therapeutic potency of NvIH, suggesting that NK cells, CD4^+^ cells, and CD8α^+^ cells are all critical to NvIH-mediated tumor therapy (fig. S21A). Last, one-fourth dose of NvIH significantly decreased the tumor therapeutic efficacy, relative to full-dose treatment, indicating the dose dependence of NvIH for tumor therapy (fig. S21B).

**Fig. 7.**
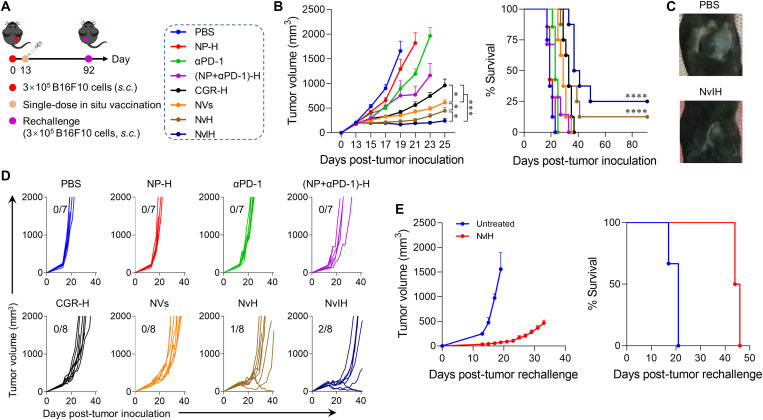
In situ vaccination with single-dose NvH and NvIH for potent and durable immunotherapy of large melanoma with immune memory. (**A**) Experimental design. C57BL/6 mice were subcutaneously inoculated with 3 × 10^5^ B16F10 cells on the right flank. On day 13, the mice were intratumorally treated with (NVs + αPD-1)-loaded in hydrogel (NvIH) or controls. (**B**) Mean tumor volumes and survival curves of as-treated B16F10 tumor-bearing C57BL/6 mice. (*n* = 7 to 8.) (**C**) Representative photographs of tumors on day 19. (**D**) Individual tumor growth curves in the above-treated mice. Fractions: complete regression rates. (**E**) Mice with complete tumor regression (*n* = 2) were rechallenged with B16F10 cells on the contralateral flank 92 days after the first tumor inoculation. Naive mice (*n* = 3) were age-matched. Data: mean ± SEM. *P* values were determined by two-way ANOVA, Tukey’s multiple-comparison test; log-rank (Mantel-Cox) test was used for animal survival comparison (*P < 0.05; ***P < 0.001; ****P < 0.0001).

We next assessed NvH and NvIH for the immunotherapy of large poorly immunogenic B16F10 melanoma (~200 mm^3^) in C57BL/6 mice (Fig. 7A). Single-dose in situ vaccination with NvH significantly decreased the tumor growth rate (DT_NvH_ = 11.2 days) relative to NVs and CGR-H (DT_NVs_ = 7.2 days, DT_CGR-H_ = 4.7 days), while blank NP-H showed no significant inhibition of tumor growth (DT_PBS_ = 1.3 days, DT_NP-H_ = 1.6 days) (Fig. 7, B and D). Combining immunostimulants with αPD-1 dramatically inhibited tumor growth [DT_NvIH_ = 17.3 days; DT_αPD-1_ = 2.3 days; DT_(NP+αPD-1)-H_ = 2.9 days] and prolonged mouse survival, with 105%, 70%, and 34% increase of median survival relative to PBS, αPD-1, and CGR-H, respectively (Fig. 7, B to D). Again, two of eight mice with complete tumor regression resisted rechallenge of B16F10 melanoma cells on the opposite flank 92 days after first tumor inoculation (Fig. 7A), with the median survival time of 45 days in contrast to 19 days of age-matched naive mice (Fig. 7E). Overall, NvH and NvIH showed robust immunotherapeutic efficacy of large tumors with potent antitumor immunity and long-lasting immune memory.

### Robust immunotherapy of large tumors with abscopal effect via systemic antitumor immunomodulation by in situ tumor vaccination with NvIH

**Fig. 8.**
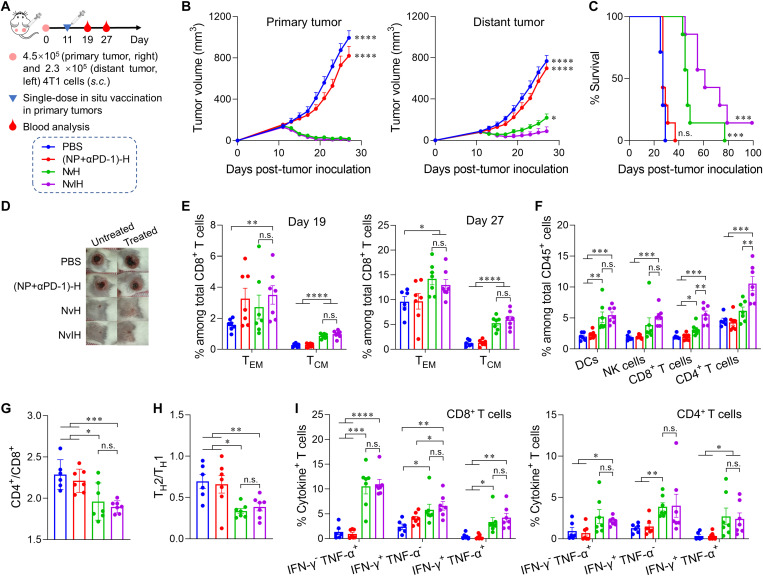
In situ tumor vaccination with single-dose NvIH for potent immunotherapy of large tumors with abscopal effect via systemic antitumor immunomodulation. (**A**) Experimental design. Balb/c mice were subcutaneously inoculated with 4.5 × 10^5^ and 2.3 × 10^5^ 4T1 cells on the right and left flanks, respectively. On day 11, the primary tumor was treated intratumorally with NvIH or controls (*n* = 7). On days 19 and 27, PBMCs were analyzed. (**B**) Tumor volume of primary (treated) and distant tumors (untreated). (**C**) Survival curve of the as-treated mice. (**D**) Representative photographs of tumors on day 27. (**E**) Percent of PBMC CD62L^−^CD44^+^CD8^+^ T_EM_ and CD62L^+^CD44^+^CD8^+^ T_CM_ on days 19 and 27. (**F**) Flow cytometric quantification of the percent of DCs (CD45^+^CD11c^+^), NK cells (CD45^+^ NK-1.1^+^), CD4^+^ T cells (CD45^+^CD3^+^CD4^+^), and CD8^+^ T cells (CD45^+^CD3^+^CD8^+^) in PBMCs on day 27. (**G**) PBMC CD4^+^/CD8^+^ T cell ratios. (**H**) Ratio of T_H_2 (CD45^+^CD4^+^IL-4^+^) to T_H_1 (CD45^+^CD4^+^IFN-γ^+^) cells in PBMCs on day 27. (**I**) Intracellular TNF-α and IFN-γ staining of PBMC CD8^+^ and CD4^+^ T cells on day 27 upon ex vivo PMA/ionomycin stimulation of T cells. For flow cytometric analysis, on day 19, *n* = 7; on day 27, *n* = 6 to 7. Data: mean ± SEM. *P* values were determined by two-way ANOVA, Tukey’s multiple-comparison test; log-rank (Mantel-Cox) test was used for animal survival comparison (n.s., not significant; *P < 0.05; **P < 0.01; ***P < 0.001; ****P < 0.0001).

Cancer metastasis is a major obstacle of cancer treatment and is the primary cause of cancer patient death (*46*). Thus, it is highly desired for in situ tumor vaccination to have effective therapy of local tumors with abscopal effect, which leverages systemic antitumor immunity for the therapy of distant tumors that can be inaccessible or inconvenient for in situ vaccination (*47*). To study this, we assessed whether in situ vaccination with single-dose NvIH induced systemic antitumor immunity for the treatment of distant tumor in Balb/c mice bearing contralateral 4T1 tumors (Fig. 8A). The primary and distant tumors were inoculated subcutaneously with 4.5 × 10^5^ and 2.3 × 10^5^ 4T1 cells, respectively. On day 11, primary tumors (135 mm^3^) received single-dose intratumoral NvIH or controls, leaving distant tumors untreated (80 mm^3^). NvH significantly inhibited the tumor growth of primary and distant tumors (distant tumor: DT_NvH_ = 14.1 days versus DT_PBS_ = 4.5 days), suggesting robust immunotherapy of large tumors with abscopal effect (Fig. 8B). NvIH further potentiated the therapeutic efficacy of distant tumors (DT_NvIH_ = 19.4 days), with 126% and 30% increase of median survival relative to (NP + αPD-1)-H and NvH (Fig. 8, B and C). Further, one of seven tumors treated with NvIH was completely regressed on day 80, with no detectable metastasis in liver (Fig. 8, C and D, and fig. S22). Hematoxylin and eosin (H&E) staining of main organs from the above treated mice showed that NvIH caused no histologically detectable toxicity to normal tissues (figs. S22 and S23). Further, no skin rash or rheumatism was observed in NvIH-treated mice (fig. S24). These results suggest the great safety potential of NvIH.

To dissect the systemic antitumor immunity underlying the above distant tumor inhibition, we analyzed PBMC immune milieu on days 19 and 27 after tumor inoculation. NvH increased the fraction of effector memory CD8^+^ T cells (T_EM_) and T_CM_ 8 days after treatment (Fig. 8E), which was further enhanced by NvIH (Fig. 8E), with 4.2- and 4.8-fold increases of T_CM_ by NvH and NvIH, respectively, relative to (NP + αPD-1)-H on day 27. Further, NvH increased the percent of NK cells, DCs, and CD4^+^ and CD8^+^ T cells (Fig. 8F and figs. S25 and S26). Remarkably, NvIH further expanded the fractions of CD4^+^ and CD8^+^ T cells (Fig. 8F), decreased the T helper 2 (T_H_2)/T_H_1 ratio, and reduced CD4^+^/CD8^+^ T cell ratios, indicating multitier systemic antitumor immunomodulation (Fig. 8, G and H). NvH and NvIH also increased the percentages of polyfunctional TNF-α/IFN-γ–secreting CD4^+^ and CD8^+^ T cells, indicating enhanced antitumor T cell quality (Fig. 8I and fig. S27). These results suggested that in situ vaccination with NvIH induced a potent systemic antitumor immunity with long-lasting memory, which is attributed to its robust immunotherapy of distant tumors.

**Fig. 9.**
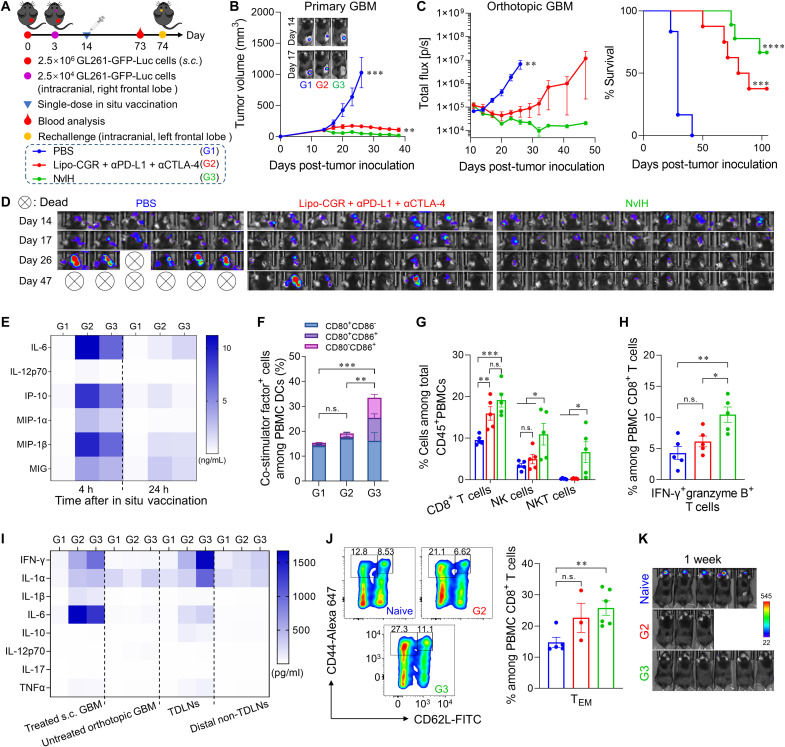
In situ tumor vaccination with single-dose NvIH for potent immunotherapy of local large peripheral GBM tumors and distant orthotopic GBM tumors with abscopal effect. (**A**) Experimental design. C57BL/6 mice were subcutaneously inoculated with 2.5 × 10^6^ GL261-GFP-Luc cells on the right flank (day 0). On day 3, 2.5 × 10^4^ GL261-GFP-Luc cells were inoculated into the frontal lobe. The subcutaneous tumor (~100 mm^3^) was intratumorally treated with single-dose (NVs + αPD-L1 + αCTLA-4)-loaded in hydrogel (NvIH) and controls on day 14. Sera were collected 4 hours, 24 hours, and 4 days after treatment for immune profiling by Luminex. (**B**) Tumor volumes and representative bioluminescence images of treated primary GL261-GFP-Luc tumors. (**C**) Total bioluminescence flux of orthotopic tumors quantified from IVIS imaging results. (**D**) Bioluminescence photographs of orthotopic tumors on days 14, 17, 26, and 47. (**E**) Serum cytokine/chemokine levels measured by Luminex 4 and 24 hours after treatment. (**F**) Percentage of CD80^+^ and/or CD86^+^ DCs in PBMCs 4 days after treatment. Data were quantified from flow cytometry results. (**G**) Percentages of CD8^+^ T, NK, and NKT cells in PBMCs on day 18. (**H**) IFN-γ^+^granzyme B^+^ CD8^+^ T cells in PBMCs on day 18. (**I**) Cytokine levels in treated local GBM (right flank), orthotopic GBM, TDLNs, and distal non-TDLNs, as quantified by Luminex 6 days after treatment. (**J**) Representative flow cytometry dot plots and quantification of PBMC CD62L^−^CD44^+^CD8^+^ T_EM_ cells on day 73 indicate that NvIH enhanced T_EM_ fractions. (**K**) Bioluminescence photographs of mice intracranially rechallenged with 2.5 × 10^4^ GL261-GFP-Luc cells in the naive left frontal lobe 1 week after challenge. Data represent mean ± SEM. *P* values were determined by two-way ANOVA, Tukey’s multiple-comparison test; log-rank (Mantel-Cox) test was used for animal survival comparison (n.s., not significant; *P < 0.05; **P < 0.01; ***P < 0.001; ****P < 0.0001).

An estimated 20% of all cancer patients develop brain metastases (*48*–*51*). Encouraged by the abscopal effect induced by in situ NvIH vaccination of local tumors, we then tested this approach for the treatment of distant brain metabasis. To mimic local and distant tumors in the case of brain metastasis, we established a dual-tumor mouse model with an orthotopic murine glioblastoma multiforme (GBM) and a subcutaneous GBM tumor on the shoulder. Current radiation therapy and chemotherapy has only limited therapeutic efficacy to brain metabases due to immunosuppressive TME and the blood-brain barrier (BBB) (*52*, *53*). Immunotherapy holds the potential to elicit systemic antitumor molecular and cellular immune responses that penetrate BBB for potent and durable therapy of GBM and brain tumor metastases (*54*). To test this, we established a dual GBM mouse model, in which subcutaneous GL261-GFP-Luc GBM was inoculated on the right flank on day 0, and an orthotopic GL261-GFP-Luc GBM was inoculated in the right frontal lobe on day 3 (Fig. 9A). On day 14, the subcutaneous tumor (~100 mm^3^) was in situ vaccinated with single-dose NvIH [i.e., (NVs + αPD-L1 + αCTLA-4)-in-hydrogel] and controls, leaving the orthotopic tumors untreated. As a result, relative to CGR-loaded liposome lipo-CGR + αPD-L1 + αCTLA-4, NvIH significantly enhanced the therapeutic efficacy of not only the treated subcutaneous GBM but also orthotopic GBM, suggesting abscopal effect (Fig. 9, B to D). Specifically, lipo-CGR + αPD-L1 + αCTLA-4 inhibited the growth of subcutaneous tumors (six of eight) but only partially inhibited the growth of the orthotopic GBM (Fig. 9, C and D, and fig. S28). By contrast, NvIH completely regressed all subcutaneous tumors and remarkably completely regressed six of nine orthotopic tumors. Overall, single-dose NvIH showed robust and durable immunotherapy for not only primary vaccinated tumors but also distant orthotopic GBM that can also mimic brain metastases.

Free molecular immunostimulants are often rapidly disseminated into blood circulation upon injection into the body, which not only compromises their therapeutic efficacy but also represents a safety concerns due to overly strong systemic inflammation, also known as cytokine storm syndrome (*55*). To assess this, we measured the serum levels of a panel of cytokines and chemokines by Luminex 4 and 24 hours after treatment, respectively. Relative to lipo-CGR + αPD-L1 + αCTLA-4, NvIH significantly reduced the serum levels of IL-6 (*P* = 0.03), IL-12p70 (*P* = 0.024), IP-10 (*P* = 0.0013), MIP-1β (*P* = 0.000022), and MIG (*P* = 0.00019) 4 hours after treatment, suggesting improved systemic tolerability of NvH that could prevent immune toxicity caused by overly strong systemic inflammation (Fig. 9E). Moreover, NvIH sustained cytokine levels for at least 4 days, likely due to the sustained release of immunostimulants and the resulting prolonged systemic antitumor immunity (fig. S29). Moreover, we analyzed PBMC immune milieu 4 days after treatment to investigate the systemic antitumor immunity underlying the abscopal effect of NvIH for the treatment of distant orthotopic GBM. Single-dose NvIH increased the fractions of matured CD80^+^ and/or CD86^+^ DCs by 2.2- and 1.8-fold relative to PBS and lipo-CGR + αPD-L1 + αCTLA-4, respectively (Fig. 9F and fig. S30, A to D). NvIH augmented the NK, NKT, and IFN-γ^+^granzyme B^+^ CD8^+^ T cell populations (Fig. 9, G and H, and fig. S30E), suggesting systemic innate (NK and NKT cells) and adaptive (CD8^+^ T cell) antitumor immunity, both critical for optimal immunotherapy.

We further examined the impact of in situ vaccination of primary tumors of NvIH on the TME immune milieu in both primary tumors and distant brain metabasis (orthotopic GBM). The above treated primary tumors, untreated orthotopic tumors, TDLNs, and distal non-TDLNs were isolated and analyzed by Luminex on day 6 after treatment. Relative to PBS, NvIH significantly increased the cytokine level in treated tumors (IFN-γ, *P* = 0.00027; IL-1α, *P* = 0.024; IL-1β, *P* = 0.013; IL-6, *P* = 0.012) and orthotopic tumors (IL-17, *P* = 0.022), suggesting the transformation of immunologically “cold” tumors to immunogenic “hot” tumors (Fig. 9I). Moreover, relative to lipo-CGR + αPD-L1 + αCTLA-4, NvIH increased IFN-γ (*P* = 0.043) level in treated tumors (Fig. 9I). IL-1β is critical for immune cell infiltration into brain tumors (*52*, *56*). Relative to PBS, NvIH elevated the levels of IL-1β by 9.79 and 0.87 times in orthotopic tumors and sera, respectively (Fig. 9I and fig. S29). Similarly, NvIH resulted in antitumor immunomodulation in TDLNs and distal non-TDLNs. Collectively, in situ tumor vaccination with NvIH remodeled tumor immune milieu and promoted TME antitumor immunity not only in the treated primary tumors but also in distant brain metabasis despite BBB.

We further studied the antitumor immune memory underlying the abscopal effect. On day 73, NvIH treatment showed 1.8- and 1.2-fold increases of T_EM_ fractions in PBMCs relative to age-matched naive mice and mice treated with lipo-CGR + αPD-L1 + αCTLA-4 (Fig. 9J). To verify the antitumor immune memory, mice with complete regression of subcutaneous and orthotopic GBMs were rechallenged by intracranial injection of GL261-GFP-Luc cells (2.5 × 10^4^ cells) into the naive left frontal lobe on day 74. As a result, NvIH completely resisted tumor rechallenge, while naive mice displayed rapid tumor growth with a short survival time (Fig. 9K and fig. S31). Meanwhile, in the above mice, NvIH did not cause any vitiligo, which often results from anti-melanocyte immunity, suggesting the potential of in situ vaccination with NvIH to elicit tumor-specific adaptive immunity (fig. S32) (*57*). These results demonstrated that NvIH elicited systemic antitumor immune memory that prevents tumor relapse.

## DISCUSSION

By educating the host immune system to elicit antitumor immunity, immunotherapy has shown great potential to treat a growing number of cancers (*1*). Various approaches of cancer immunotherapy have been studied, ranging from adoptive antitumor immune cell transfer, ICB, to therapeutic vaccines, alone or in combination (*2*, *4*, *8*, *9*). ICB is one of the most successful approaches in the clinic thus far (*1*). However, overall, only a small subset of cancer patients benefits from current ICB therapy, largely due to multitier immunosuppression in the TME. In situ vaccination intrinsically delivers multivalent antitumor immunostimulants, allowing to reduce the immunosuppression and enhance the antitumor immunity in TME (*24*–*27*). In situ vaccination with various immunostimulants, such as TLR9 agonist CpG, TLR3 agonist poly-lysine and carboxymethylcellulose, Fms-like tyrosine kinase 3 ligand, IL-2, and IL-12, has been tested in preclinical and clinical trials (*28*–*30*). Yet, most of these immunostimulants have thus far shown suboptimal therapeutic efficacy due to a number of complications such as poor tumor retention and the resulting toxic acute off-targeting immunostimulation upon rapid systemic drug dissemination, and the lack of effective presentation of a broad spectrum of bona fide tumor neoantigens and TAAs (*30*–*34*).

Here, we developed NvIH using injectable NPs-in-hydrogel composites that were loaded with triple immunostimulants and ICBs for prolonged tumor retention and controlled release of these immunotherapeutics, multitier innate and adaptive antitumor immunostimulation in TME and systemically, and robust cancer immunotherapy of large local and distant tumors with abscopal effect in multiple tumor models. Specifically, we first synthesized a series of cationic polymers and selected PHD-AEP as the nanocarrier for these molecular immunostimulants. PHD-AEP self-assembled into NPs with a hydrophobic core for R848 loading and a brush cationic surface for the loading of anionic CpG and cGAMP. PHD-AEP NPs enhanced the intracellular co-delivery of immunostimulants to elicit potent antitumor immunostimulation. Next, the thermoresponsive injectable hydrogel used in NvIH allows for surgery-free minimally invasive syringe injection, followed by fast gelation at physiological temperature that prolongs tumor retention and sustains drug release from the NvIH reservoir. Moreover, the triple immunostimulants of TLR7/8/9 agonists and STING agonist elicited potent proinflammatory responses and are expected to expand the activatable immune cell subsets that have differential expression profiles of TLR7/8/9 and STING. Together, NvIH formulation prolonged the tumor retention of immunostimulants and reduced potentially toxic acute systemic inflammation associated with systemic immunostimulant dissemination. Remarkably, in situ tumor vaccination with single-dose NvIH reduced TME immunosuppression, increased tumor antigen accumulation in TDLNs, and elicited potent systemic antitumor T cell responses with memory, the latter of which is attributed to the abscopal effect shown in the therapy of multiple types of tumors. Relative to NvH, NvIH notably potentiated the tumor therapeutic efficacy and enhanced the fractions of tumor complete regression. Particularly, single-dose NvIH inhibited or completely regressed not only the vaccinated large tumors but also distant large tumors, in multiple syngeneic mouse models with poorly immunogenic tumors (4T1 mammary carcinoma in Balb/c mice, B16F10 melanoma in C57BL/6 mice, and GL261-GFP-Luc GBM in C57BL/6 mice). Worth noting, due to the systemic antitumor immunity and abscopal effect, single-dose NvIH treatment in peripheral primary tumors inhibited or eradicated not only distant peripheral tumors but also distant metastasis-mimicking orthotopic GBM despite BBBs. This provides a potential avenue to minimally invasive brain cancer immunotherapy by eliciting systemic antitumor immunity, which is highly desired to treat brain cancers. Moreover, in mice with complete tumor eradiation, the antitumor immune memory enabled these mice to resist tumor challenges, which is expected to prevent tumor recurrence and enhance the long-term survival rates.

Overall, NvIH holds great potential for the combination immunotherapy of many types of local or metastatic solid cancers that are surgically accessible for in situ vaccination. Single-dose NvIH already showed robust tumor therapeutic efficacy, likely due to the efficient tumor retention of three potent synergistic immunostimulants as well as ICB antibodies, and presumably the lack of anti-carrier immunity that allows prolonged tumor retention of NvIH and sustained drug release from the NvIH reservoir. Further studies are needed to test our hypothesis that these synthetic biomaterial-based NvIH bypasses preexisting host anti-carrier immunity, and then test whether NvIH allows repeated dosing, which can further enhance the tumor therapeutic efficacy and increase the complete regression rate of tumor. Furthermore, in situ tumor vaccination has shown great potential for tumor immunotherapy. Nonetheless, in situ vaccination may find limitations in tumors that are not surgically accessible, including blood cancer, many types of solid cancers, as well as non-operable cancers such as deadly diffuse intrinsic pontine glioma (DIPG). Last, comprehensive short-term and long-term toxicology studies are needed to evaluate the safety of NvIH, especially if repeatedly dosed, which likely elevates immune-related toxicity.

## MATERIALS AND METHODS

### Cell culture

RAW264.7 macrophages, B16F10 cells, and GL261-GFP-Luc cells were cultured in Dulbecco’s modified Eagle’s medium (DMEM). DC2.4 and 4T1 cells were cultured in RPMI 1640 medium. All medium was supplemented with 10% fetal bovine serum (Gibco), penicillin (100 U/ml), and streptomycin (100 μg/ml). RAW-Lucia ISG cells were purchased from InvivoGen and cultured following the specifications. All cells were grown in a humidified atmosphere (5% CO_2_, 37°C).

### Polymer synthesis and screening for vaccine delivery

#### Materials

AEP, dimethyl sulfoxide-*d*_6_ (DMSO-*d*_6_), *p*-toluenesulfonyl chloride (TsCl), ammonia solution (28 to 30%), DAB, triethylamine (TEA), and TEPA were purchased from Sigma-Aldrich. Dithiothreitol (DTT) was purchased from Fisher Scientific. 1,6-Hexanediol diacrylate (HDA) was purchased from Alfa Aesar.

#### Synthesis of PHD

PHD was synthesized using thiol-Michael addition polymerization as previously described. HDA (2.00 g) and DTT (1.36 g) were dissolved in DMSO and purged with Ar for 30 min. Then, TEA (0.09 g) was added and polymerized at room temperature for 48 hours. After that, the polymer was dialyzed against water for 48 hours and freeze-dried. The polymers were characterized by ^1^H NMR (Bruker UltraShield 400 Plus) in DMSO-*d*_6_ and GPC (Waters 2414).

#### Synthesis of PHD-NH_2_, PHD-DAB, PHD-AEP, and PHD-TEPA

PHD (3.00 g) and TsCl (7.50 g) were dissolved in dimethylformamide (DMF) and purged with Ar for 30 min. Then, TEA (2.40 g) was added dropwise and further reacted for 24 hours at 4°C. The mixture was precipitated three times in cold diethyl ether and vacuum-dried at room temperature. The purified polymer was dissolved in DMF and then added to an ammonia solution (28 to 30%), DAB, AEP, or TEPA and reacted at room temperature for 4 hours. Subsequently, the mixture was dialyzed against water for 72 hours, and PHD-NH_2_, PHD-DAB, PHD-AEP, or PHD-TEPA was obtained after freeze-drying. The polymers were characterized by ^1^H NMR.

#### Screening vaccine delivery polymers

Solvent evaporation and physical complexation were used to fabricate blank NPs and cGAMP-loaded NPs. Take PHD-AEP as an example, PHD-AEP (1 mg) was dissolved in 200 μl of tetrahydrofuran (THF) and added dropwise to deoxyribonuclease (DNase)– and ribonuclease (RNase)–free H_2_O. The mixture was stirred at room temperature overnight to evaporate THF and centrifuged (4000 rpm, 5 min) to get blank PHD-AEP NPs. Then, the size, zeta potential, and cytotoxicity of blank NPs were characterized by Zetasizer (Malvern Nano ZS90) and 3-(4,5-dimethylthiazol-2-yl)-2,5-diphenyltetrazolium bromide (MTT) assay, respectively.

Subsequently, the binding ability of nucleic acid (CpG and cGAMP) and NPs was verified. Briefly, we first evaluated in vitro cGAMP cellular uptake and activity. For cGAMP cellular uptake, CDG^DY547^ (G; Biolog) was used as a mimetic molecule of cGAMP. CDG^DY547^ was mixed with blank NPs with different N:P ratios (50, 25, and 12) to prepare different G/NP complexes. Then, these complexes and free CDG^DY547^ were added to a 12-well plate seeded with RAW264.7 cells (0.2 × 10^6^ cells per well) and cultured for 1 hour, and the relative cellular uptake was quantified by flow cytometry (BD FACSCanto II). For cGAMP (2′3′-cGAMP; G; InvivoGen) activity study, RAW-Lucia ISG cells were seeded in a 96-well plate (0.03 × 10^6^ cells per well) and cultured for 24 hours. G/NP complexes (N:P = 50) were prepared as previously described. Then, free cGAMP and G/NP complexes were added to the plate and further cultured for 24 hours. The relative production of IFN-α/β in the medium was quantified with QUANTI-Luc (InvivoGen).

### Fabrication and characterization of NVs

Solvent evaporation and physical complexation were used to fabricate NVs. First, PHD-AEP (5 mg) and R848 (0.55 mg; Sigma-Aldrich) mixed with 1 ml of THF were dropwise added to DNase- and RNase-free H_2_O. The mixture was stirred at room temperature overnight to evaporate THF and centrifuged (4000 rpm, 5 min) to remove free R848 to get R848-loaded NPs (R/NPs). Subsequently, R/NPs and cGAMP (G) were mixed and incubated for 30 min at room temperature to prepare GR/NPs. GR/NPs were purified by ultrafiltration (30-kDa cutoff; Millipore). The concentrations of R848 and cGAMP were tested by high-performance liquid chromatography (HPLC) (SHIMADZU). Finally, GR/NPs were mixed with CpG (CpG ODN 1826; IDT) and incubated for 30 min at room temperature to prepare NVs. The size, zeta potential, stability, and morphology were characterized with Zetasizer (Malvern Nano ZS90) and a transmission electron microscope (JEOL JEM-1400).

### NvH preparation and drug release

Thermosensitive injectable NvH (25 wt % gel) was prepared via physical mixing of Pluronic F-127 (12600 g/mol; Sigma-Aldrich) and NVs. Briefly, 500 μl of NVs and 125 mg of F-127 were placed in the small glass vial. Then, the vial was vortexed and placed on the ice to prepare NvH. To study drug release, NVs and NvH (150 μl) were placed in mini-dialysis tubes (molecular weight cutoff 20,000, Thermo Fisher Scientific) and immersed in tubes with different release medium (1 ml, pH 6.6 and 7.4). The tubes were placed in a shaker (120 rpm, 37°C), 0.1 ml of release medium was taken out at different time points, and 0.1 ml of fresh release medium was added. The concentrations of R848 and cGAMP were measured by HPLC.

### In vitro evaluation of cell uptake and APC activation

#### Cell uptake

CDG^Fluo^ (G; Biolog) and CpG^AF594^ (C; IDT) were used as fluorescence probes. Fluorescently labeled CpG and CDG co-loaded NPs (CG/NPs) were prepared as previously described. RAW264.7 cells were seeded in 12-well plates (0.2 × 10^6^ cells per well) and cultured for 24 hours. Then, fluorescently labeled CDG + CpG (CG; physical mixing) and CG/NPs were added and further cultured for 0.5, 1, and 2 hours. CDG^Fluo^ and CpG^AF594^ concentration was 0.5 μg/ml and 50 nM, respectively. The cells were harvested, washed, and detected using flow cytometry (BD LSRFortessa X-20). For confocal microscopy observation, DC2.4 cells were seeded in glass dishes and cultured overnight. Then, CG or CG/NPs were added and further cultured for 2 hours. Cells were washed with PBS, stained with Hoechst 33342, fixed, and imaged (Zeiss LSM 710).

#### Proinflammatory factors

RAW264.7, DC2.4, and RAW-Lucia ISG cells were seeded in 96-well plates (0.03 × 10^6^ cells per well) and cultured for 24 hours. Then, CpG, R848, cGAMP, CGR, and NVs were added and further cultured for 24 hours. The concentrations of CpG, R848, and cGAMP were 50 nM, 150 nM, and 1 μg/ml, respectively. For RAW264.7 and DC2.4 cell lines, the concentrations of IL-6, IL-12p40, and TNF-α in the medium were quantitated by enzyme-linked immunosorbent assay (ELISA; R&D Systems). For the RAW-Lucia ISG cell line, the relative production of IFN-α/β in the medium was quantified with QUANTI-Luc (InvivoGen).

#### Expression of costimulatory factors

DC2.4 cells were seeded in a six-well plate (0.5 × 10^6^ cells per well) and cultured for 24 hours. Then, CpG, R848, cGAMP, CGR, and NVs were added and further cultured for 24 hours. The cells were collected and stained with αI-A/I-E, αCD40, αCD80, and αCD86 antibodies (BioLegend). The cells were washed, and the expression levels of costimulatory factors were detected by flow cytometry (BD FACSCanto II).

#### Long-term activation by NvH

RAW264.7 cells were seeded in 24-well transwell plates (0.1 × 10^6^ cells per well, 0.5-ml medium per well) and cultured for 24 hours. Then, CGR, NVs, and NvH (150 μl) were added to the transwell. The amounts of CpG, R848, and cGAMP in each transwell were 0.05 nmol, 0.15 nmol, and 1 μg, respectively. At set time points, transwells were taken out and put in a new 24-well plate with RAW264.7 cells. The concentrations of TNF-α and IL-12p40 in the medium were quantitated by ELISA.

### Animal studies

All work conducted on animals was in accordance with a protocol approved by the Institutional Animal Care and Use Committee (IACUC) of Virginia Commonwealth University. Female C57BL/6 and Balb/c mice (6 weeks) were purchased from Charles River Laboratories. Mice were randomly assigned to different groups. For intratumoral administration, the hydrogel-related drug formulations in 1-ml syringes with 27-gauge needles (Fisher Scientific) were placed on the ice, and then drug formulations were intratumorally injected slowly.

### In vivo drug retention

IR800-labeled CpG (IR800-CpG; IDT), DIR (D, Thermo Fisher Scientific), and CDG^DY547^ (G) were used. IR800-CpG loaded NvH (NvH^IR800^), DIR-loaded NvH (NvH^DIR^), and CDG^DY547^-loaded NvH (NvH^DY547^) were prepared as described above. 4T1 cells (4.5 × 10^5^) were subcutaneously injected into the right flank of Balb/c mice. Once reaching approximately 150 mm^3^, IR800-CpG, IR800-CpG-H, and NvH^IR800^ (100 μl) containing 0.3 nmol of IR800-CpG were intratumorally injected. Fluorescence was monitored on the IVIS Spectrum Preclinical In Vivo Imaging System (PerkinElmer IVIS Spectrum). Mice were sacrificed on day 11, and fluorescence intensities were assessed in main organs and tumors. Similarly, NvH^DIR^ (DIR: 0.5 mg/kg) and NvH^DY547^ (CDG^DY547^: 0.5 nmol) were studied.

### Antigen capture and delivery

#### In vitro antigen capture and delivery

OVA was used as a model antigen. Different concentrations of NPs (50 μl) were added to the OVA solution (50 μl, 0.5 mg/ml) and incubated at 37°C for 30 min. After that, the mixture was centrifuged (12,000*g*, 10 min), and the supernatant was collected. The concentration of supernatant was detected with the Micro BCA Protein Assay Kit (Thermo Fisher Scientific). To evaluate the antigen cross-presentation ability of NPs, DC2.4 cells were seeded in 24-well plates (0.15 × 10^6^ cells per well) and cultured for 24 hours. Then, free OVA and OVA/NP complexes (OVA concentration of 5 μg/ml) were added and further cultured for 24 hours. Cells were collected and stained with the APC anti-mouse H-2K^b^ bound to SIINFEKL antibody (BioLegend) before washing and assessment by flow cytometry (BD FACSCanto II).

#### In vivo antigen capture, delivery, and antigen-specific T cell response

OVA^AF647^ was used for in vivo study. First, 4T1 cells (4.5 × 10^5^) were subcutaneously injected into the right flank of Balb/c mice. On day 11, 50 μl of OVA^AF647^ (50 μg) was injected intratumorally. After 10 min, 100 μl of PBS or NvH was injected intratumorally at the location of OVA^AF647^ injection. After 24 hours, the distribution of OVA^AF647^ in tumor and inguinal lymph nodes was monitored by IVIS, and the fluorescence intensity of OVA^AF647^ in inguinal lymph node DCs was characterized by flow cytometry (BD LSRFortessa X-20). To study the antigen-specific T cell response, 50 μl of OVA (50 μg) was injected intratumorally into 4T1 tumor–bearing Balb/c mice. After 10 min, 100 μl of CGR or NvH was injected intratumorally at the location of OVA injection. At 4 days after treatment, PBMCs were collected and analyzed by flow cytometry (BD LSRFortessa X-20).

### Tumor immunotherapy

For single tumor models, B16F10 cells (3 × 10^5^) and 4T1 cells (4.5 × 10^5^) were subcutaneously injected into the right flank of C57BL/6 and Balb/c mice, respectively. The tumor was monitored every 2 days, and size was calculated using the following equation: *V* = *ab*^2^/2 (*a*: length; *b*: width). Once reaching about 200 mm^3^ (B16F10) and 150 mm^3^ (4T1), tumors were treated intratumorally with different drug formulations containing 200 μg of CpG, 100 μg of R848, 10 μg of cGAMP, and 200 μg of αPD-1. Mice were euthanized when tumor volume reached 2000 mm^3^. For tumor rechallenge studies, B16F10 cells (3 × 10^5^) and 4T1 cells (4.5 × 10^5^) were subcutaneously injected into the opposite flank of C57BL/6 and Balb/c mice, respectively. Tumor volume was monitored every 2 days. For antibody-mediated lymphocyte depletion, antibodies (αCD8a, αCD4, αNK1.1) were intraperitoneally injected 4 days before treatment and every 3 days thereafter and for a total of five times.

For bilateral 4T1 tumor model, 4.5 × 10^5^ and 2.3 × 10^5^ 4T1 cells were subcutaneously injected into the right and left flanks of Balb/c mice, respectively. On day 11 after tumor inoculation, tumors were treated intratumorally as previously described (CpG: 200 μg; R848: 100 μg; cGAMP: 10 μg; αPD-1: 200 μg). On days 19 and 27, PBMCs were collected and analyzed by flow cytometry (BD LSRFortessa X-20). Mice were euthanized when tumor volume reached 2000 mm^3^. For the GBM orthotopic and subcutaneous bilateral tumor model, 2.5 × 10^6^ GL261-GFP-Luc cells were subcutaneously injected into the right flank of C57BL/6 mice (day 0). Three days later, the orthotopic GBM tumors were established by injecting 2.5 × 10^4^ GL261-GFP-Luc cells into the frontal lobe (day 3). Mice were imaged using the IVIS Spectrum Preclinical In Vivo Imaging System (PerkinElmer IVIS Spectrum) to monitor tumor growth every 3 days. On day 14, tumors on the right flank (~100 mm^3^) were treated with PBS (*n* = 6), lipo-CRG + αPD-L1 + αCTLA-4 (*n* = 8), and NvIH (*n* = 9), while orthotopic tumors did not receive any treatment (CpG: 200 μg; R848: 100 μg; cGAMP: 10 μg; αPD-L1: 200 μg; αCTLA-4: 200 μg). The subcutaneous tumor volume was monitored every 3 days and calculated using the equation described previously. Mice were euthanized when subcutaneous tumor volume reached 1500 mm^3^ or when body weight loss surpassed 20%.

### Analysis of 4T1 tumor TME and systemic immune response

For tumor TME analysis, 4T1 tumors were established and treated intratumorally with different drug formulations containing 200 μg of CpG, 100 μg of R848, 10 μg of cGAMP, and 200 μg of αPD-1. Mice were sacrificed on day 4 after treatment, and the tumors and spleens were harvested. Tumors were weighed and dissociated with ophthalmic scissors, and then some tumors were digested in a solution containing collagenase (Sigma-Aldrich) and DNase I (New England Biolabs) in RPMI 1640 medium (15 min, 37°C). Subsequently, tumors were strained through a 70-μm cell strainer (Fisher Scientific) and treated with ACK Lysing Buffer (Gibco). For the systemic immune response study, spleens were strained through a 70-μm cell strainer directly and the follow-up process was the same as for tumors. Cells were washed twice with PBS, collected, and stained with antibodies according to the manufacturer’s specifications. For intracellular cytokine (IFN-γ, TNF-α, IL-4) staining, cells were restimulated with phorbol 12-myristate 13-acetate (PMA)/ionomycin/brefeldin A. Samples were assessed using flow cytometry (BD LSRFortessa X-20).

For qPCR analysis, the total RNA of harvested tumor (4 days after treatment) was isolated using the PureLink RNA Mini Kit (Thermo Fisher Scientific), and the High-Capacity cDNA Reverse Transcription Kit (Thermo Fisher Scientific) was used for RNA reverse transcription. qPCR analysis was performed using Power SYBR Green PCR Master Mix (Thermo Fisher Scientific) and forward-reverse primer pairs as described before (*8*).

### Luminex analysis

For cytokine/chemokine analysis by Luminex, sera were collected 4 hours, 24 hours, and 4 days after treatment, and subcutaneous and orthotopic tumors and TDLNs and distal non-TDLNs were collected on day 6 after treatment. Tumor and lymph node lysis was performed with T-PER tissue protein extraction reagent (Thermo Fisher Scientific) with 1% Halt protease and phosphatase inhibitor cocktail (Thermo Fisher Scientific). The lysates were centrifuged to remove debris, and the supernatant was subsequently filtered using Corning Costar Spin-X centrifuge tube filters. The sera and cell lysates were analyzed at the University Virginia Flow Cytometry Core for Luminex analysis (mouse innate immune response panel and mouse proinflammatory cytokine panel).

### Flow cytometry

Data were analyzed with FlowJo software. Antibodies to mouse CD45 (catalog no. 103133 and 103108), CD3 (catalog no. 100210), CD4 (catalog nos. 100434 and 100446), CD8α (catalog no. 100714), CD86 (catalog nos. 105008 and 105026), CD11c (catalog no. 117346), CD11b (catalog no. 101210), CD206 (catalog no. 141721), CD25 (catalog no. 101904), CD62L (catalog no. 104406), CD44 (catalog no. 103018), CD80 (catalog no. 104718), CD40 (catalog no. 102906), F4/80 (catalog no. 123118), Ly-6G/Ly-6C (catalog no. 108417), FOXP3 (catalog no. 126408), NK-1.1 (catalog no. 108710), I-A/I-E (catalog no. 107608), IFN-γ (catalog no. 505830), TNF-α (catalog no. 506308), IL-4 (catalog no. 504124), granzyme B (catalog no. 515406), and CD16/32 (catalog no. 101320) were obtained from BioLegend. Phycoerythrin-conjugated tetramer was from National Institutes of Health (NIH) Tetramer Core Facility. All reagents were used according to the manufacturer’s specifications.

### Statistical analysis

Data are presented as mean ± SD or mean ± SEM. Statistical significance was analyzed by one-way or two-way analysis of variance (ANOVA), followed by Tukey’s multiple-comparison test unless denoted otherwise (Microsoft Excel). Log-rank (Mantel-Cox) test was used for animal survival comparison (GraphPad Prism 9). *P* < 0.05 was considered statistically significant (n.s., not significant; *P < 0.05; **P < 0.01; ***P < 0.001; ****P < 0.0001; *****P < 0.00001).

## Notes

View/request a protocol for this paper from *Bio-protocol*.
