# Supplementary material for: Single-dose injectable nanovaccine-in-hydrogel for robust immunotherapy of large tumors with abscopal effect
Source: Sci Adv. 2023 Jul 14;9(28):eade6257. doi: 10.1126/sciadv.ade6257 (PMC10348685; doi:10.1126/sciadv.ade6257)
Supplement: Supplementary file 1 — Figs. S1 to S32 [file sciadv.ade6257_sm.pdf]

Science Advances
AAAS

Supplementary Materials for

Single-dose injectable nanovaccine-in-hydrogel for robust immunotherapy of large tumors with abscopal effect

Furong Cheng *et al.*

Corresponding author: Shuibin Lin, linshb6@mail.sysu.edu.cn; Weisheng Guo, tjuguoweisheng@126.com; Guizhi Zhu, guizhiz@umich.edu

Sci. Adv. **9**, eade6257 (2023)
DOI: 10.1126/sciadv.ade6257

This PDF file includes:

Figs. S1 to S32

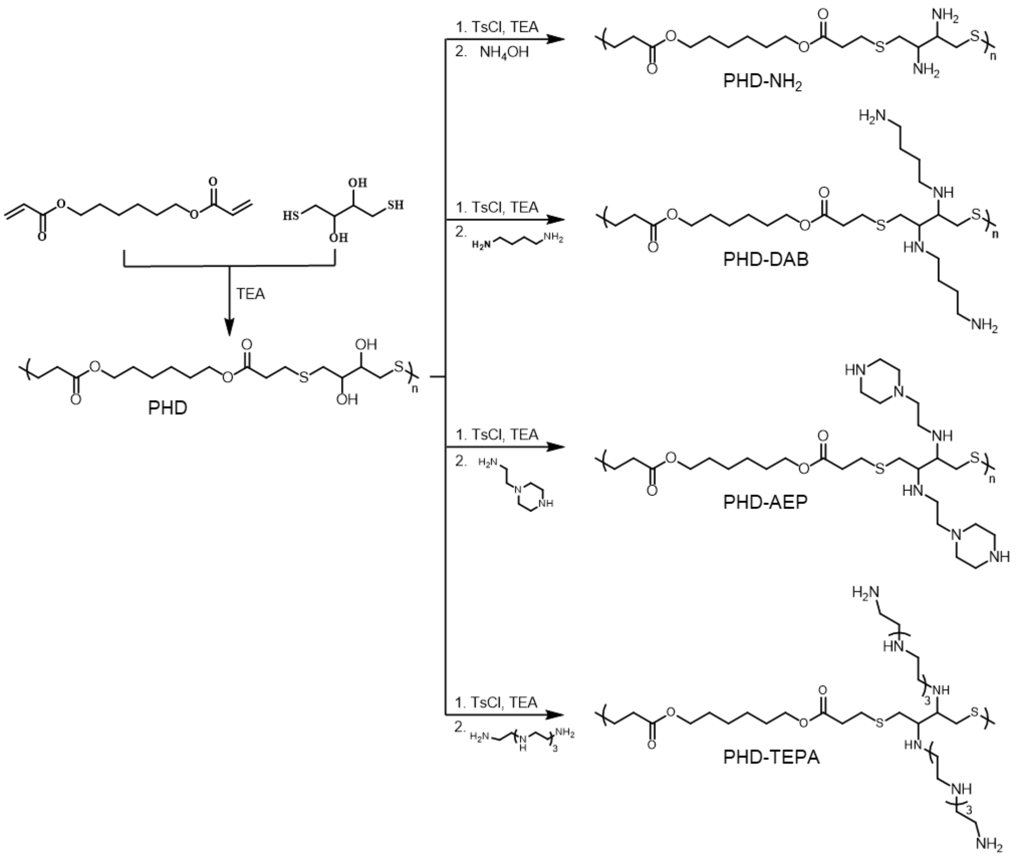

Figure S1. Synthetic routes of a series of cationic polymers.

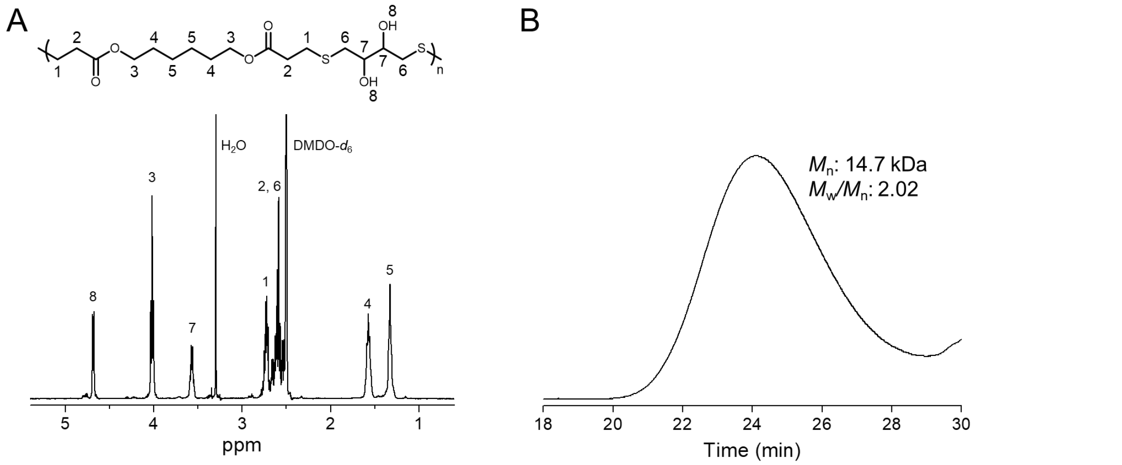

Figure S2. Characterization of polymer PHD. (**A**) ^{1}H NMR (DMSO-d_6) and (**B**) GPC spectra of PHD with THF as the eluent (1 mL/min), and the molecular weight (Mn) and M_w/M_n were obtained with polystyrene as the standard.

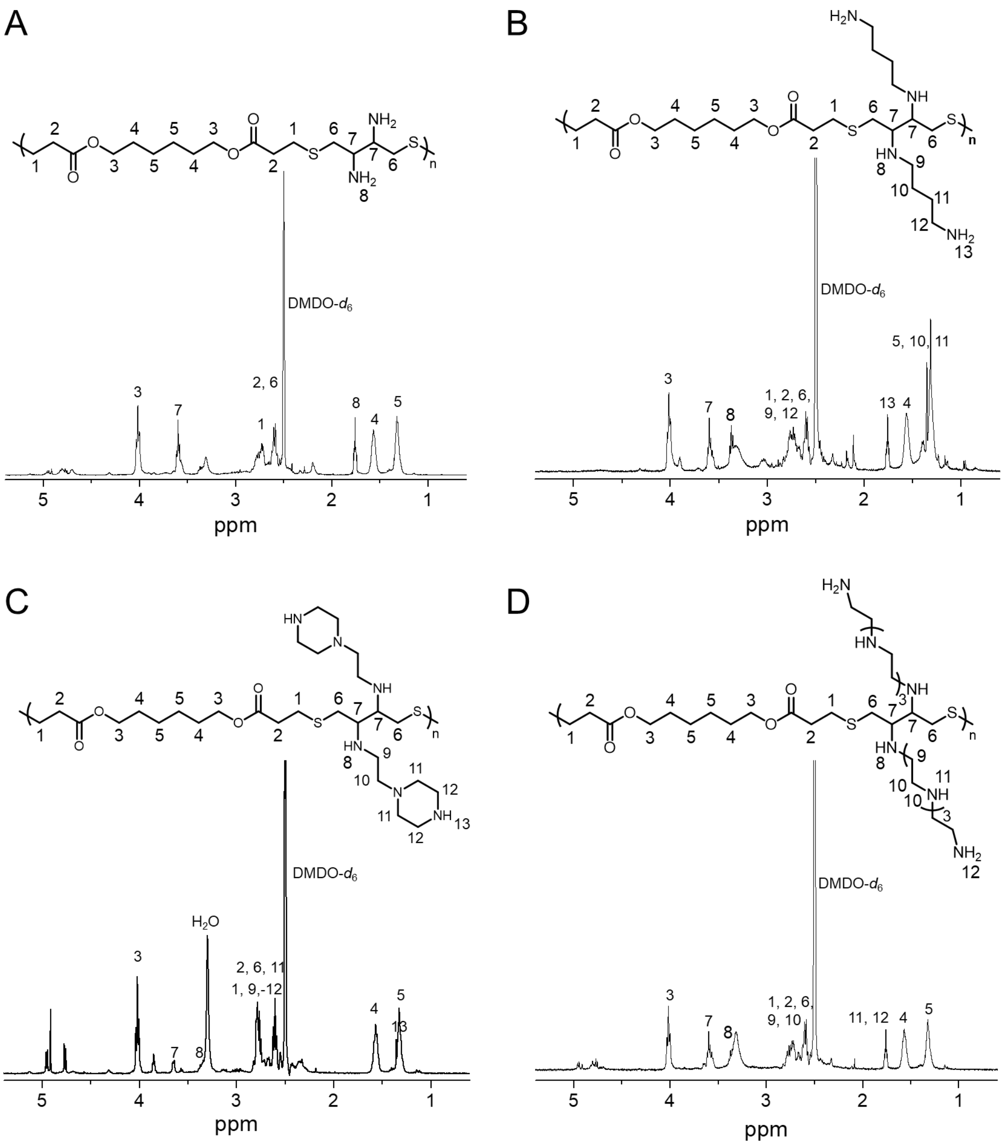

Figure S3. Characterization of candidate polymers for nanocarriers. ^1H NMR spectra (in DMSO-d_6) of polymer screened for the delivery of immunostimulants. (**A**) PHD-NH_2; (**B**) PHD-DAB; (**C**) PHD-AEP; (**D**) PHD-TEPA.

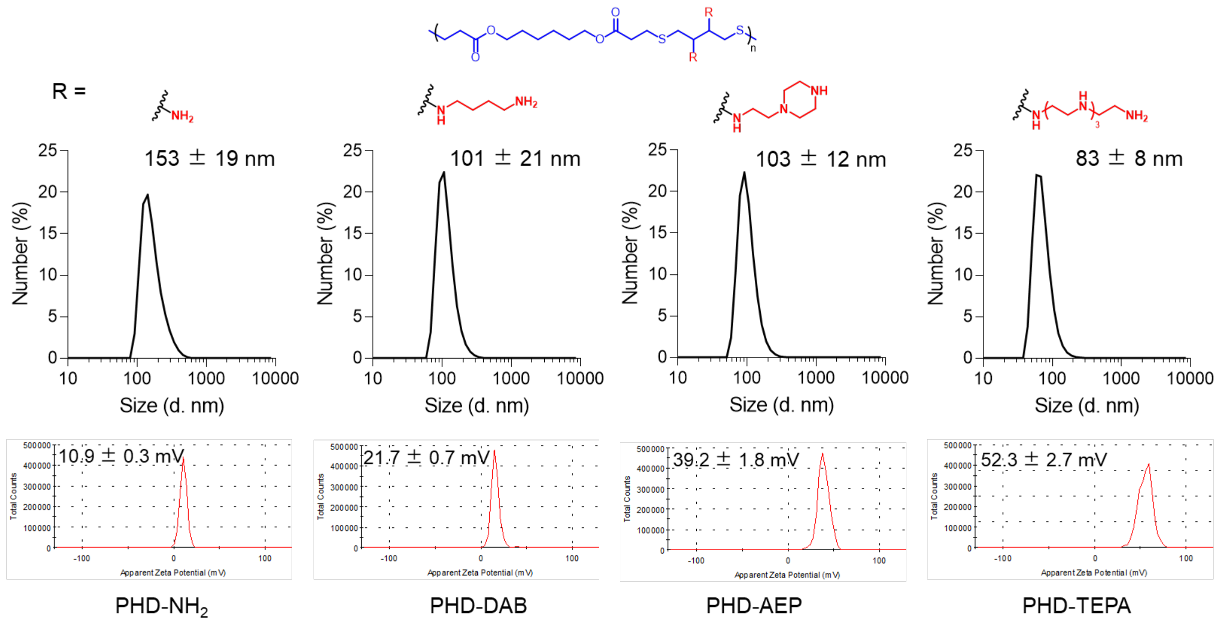

Figure S4. Characterization of candidate polymeric nanocarriers. Hydrodynamic particle sizes and zeta potentials of polymer nanoparticles. The concentration of nanoparticles was 1 mg/mL.

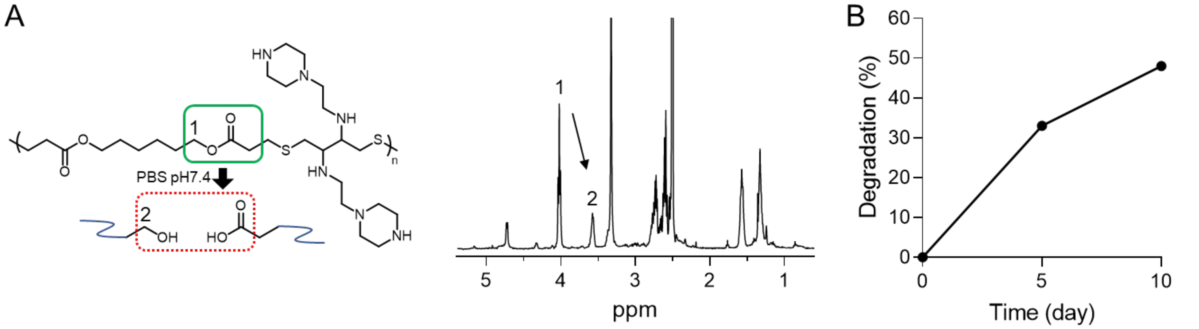

Figure S5. Degradation of PHD-AEP NPs studied by ^{1}H NMR. (**A**) The hydrolysis site (left) and ^{1}H NMR spectra after incubation of PHD-AEP NPs in PBS (pH 7.4, 37 °C) for 5 days. (**B**) Degradation of PHD-AEP NPs.

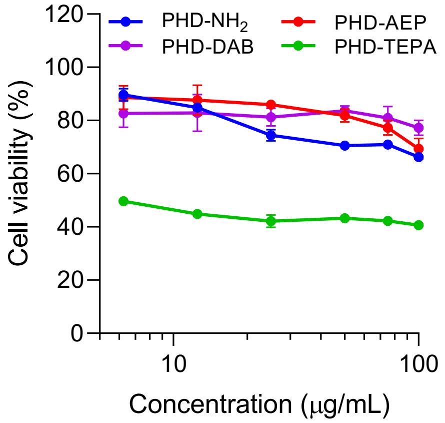

Figure S6. Cell viability of polymer nanoparticles in RAW264.7 murine macrophages. Treatment time: 24 h. Data: mean ± SD.

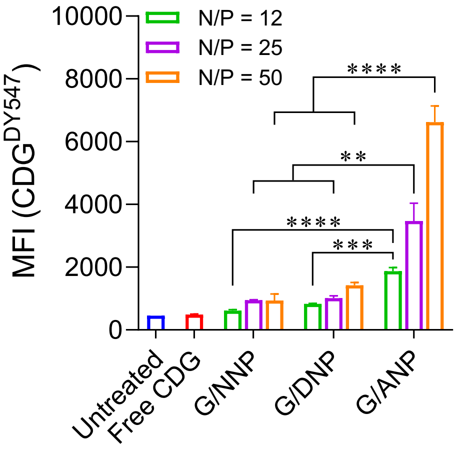

Figure S7. *In vitro* uptake of CDG delivered by NPs at different N/P ratios in macrophages. Mean fluorescence intensities (MFI), as quantified from flow cytometric results, of CDG^{DY547} (G) in RAW264.7 macrophages after incubation with G/NP complexes with different N/P ratios for 1 h (n = 3). PHD-NH_2 NPs: NNP; PHD-DAB NPs: DNP; PHD-AEP NPs: ANP. Data: mean ± SD. P values were determined by two-way ANOVA, Tukey's multiple comparison test (**$p < 0.01$; ***$p < 0.001$; ****$p < 0.0001$).

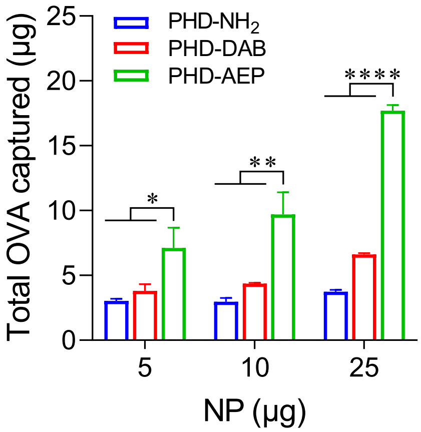

Figure S8. Total OVA captured by NPs. OVA was incubated with different concentrations of NPs (n = 3). Data: mean ± SD. P values were determined by two-way ANOVA, Tukey's multiple comparison test (*$p < 0.05$; **$p < 0.01$; ****$p < 0.0001$).

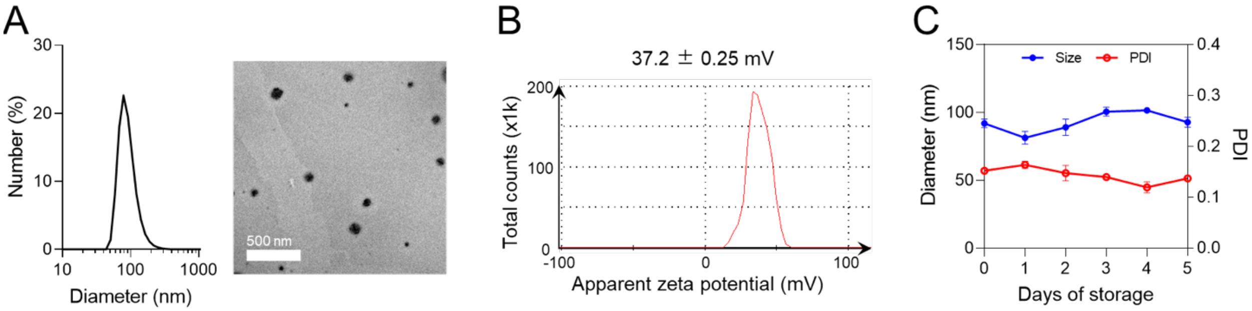

Figure S9. **Characterization of R848 loading into PHD-AEP NPs.** (**A**) Hydrodynamic particle size distribution of R848-loaded NPs (R/NPs); the inset shows a TEM image of R/NPs. (**B**) Zeta potential of R/NPs. (**C**) The stability of R/NPs in water (1 mg/mL, ambient temperature).

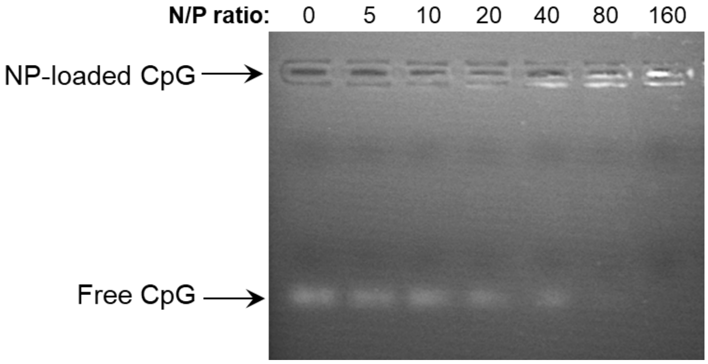

Figure S10. **Characterization of CpG loading into PHD-AEP NPs.** Gel retardation assay of free CpG and CpG-loaded NPs with a series of N/P ratios.

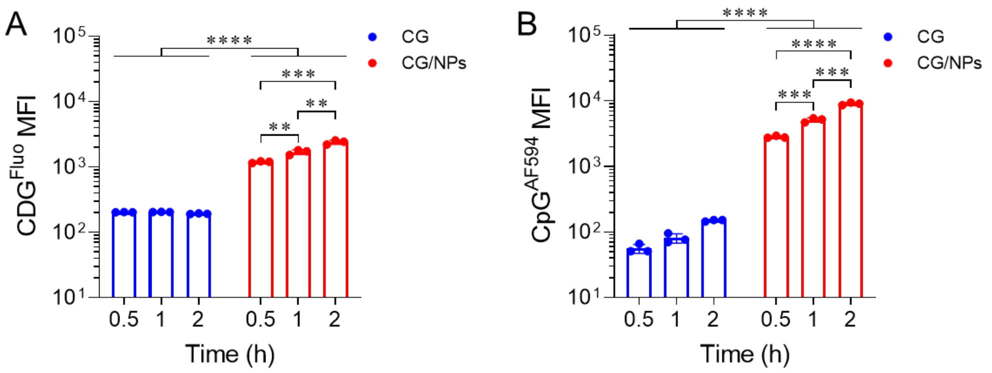

Figure S11. ***In vitro* cell uptake of CDG and CpG delivered by NPs.** The MFI, as quantified from flow cytometric results, of CDG^{Fluo} (**A**) or CpG^{AF594} (**B**) in RAW264.7 macrophages after incubation with mixed free CDG^{Fluo} + CpG^{AF594} (CG) or CG-loaded NPs (CG/NPs) for 0.5, 1, and 2 h (n = 3). Data: mean ± SD. P values were determined by two-way ANOVA, Tukey's multiple comparison test (**$p < 0.01$; ***$p < 0.001$; ****$p < 0.0001$).

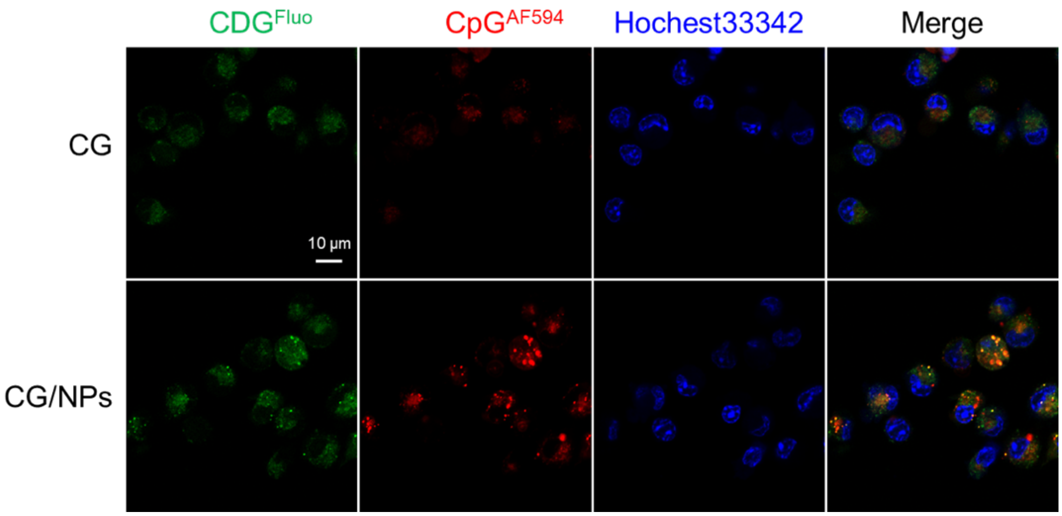

Figure S12. **Confocal microscopy imaging of the intracellular delivery of NP-delivered immunostimulants.** Shown are fluorescein-labeled CDG (CDG^{Fluo}) (0.5 μg/mL) and CpG^{AF594} (50 nM) delivered by NPs, in comparison to free CDG^{Fluo} + CpG^{AF594} (CG), in cultured DC2.4 cells (treatment: 2 h). Hochest 33342 stains nuclei.

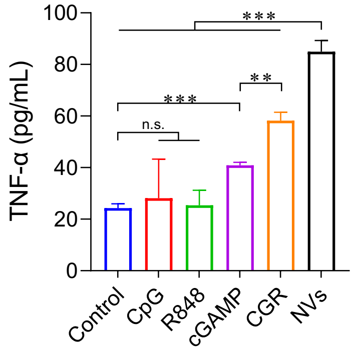

Figure S13. **ELISA results showing TNF-α concentration in the medium of as-treated DC2.4 cells** (CpG: 50 nM; R848: 150 nM; cGAMP: 1 μg/mL; treatment: 24 h; $n = 3$). Data: mean ± SD. P values were determined by two-way ANOVA, Tukey's multiple comparison test (*n.s.*: not significant; $**p < 0.01$; $***p < 0.001$).

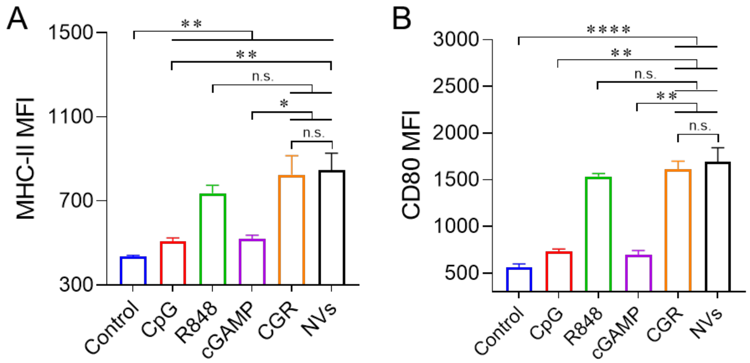

Figure S14. **NVs promoted DC maturation *in vitro*.** MFI of MHC-II (**A**) and costimulatory factor CD80 (**B**) on as-treated DC2.4 cells. CGR: CpG + cGAMP + R848. Data were quantified from flow cytometry results. Data: mean ± SD. P values were determined by two-way ANOVA, Tukey's multiple comparison test (*n.s.*: not significant; $*p < 0.05$; $**p < 0.01$; $****p < 0.0001$).

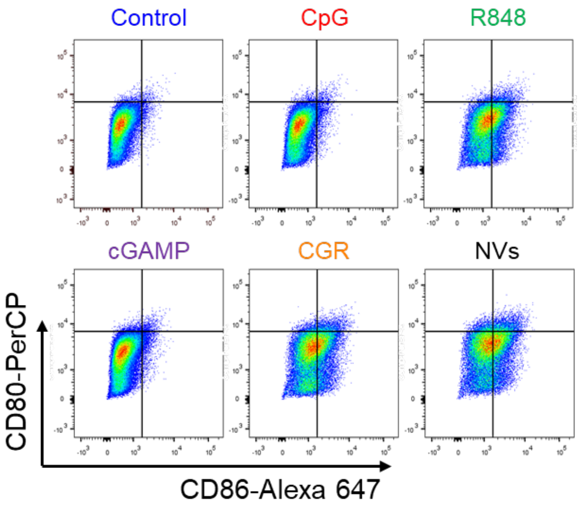

Figure S15. **Representative flow cytometry dot plots of CD80 and CD86 staining on as-treated DC2.4 cells.**

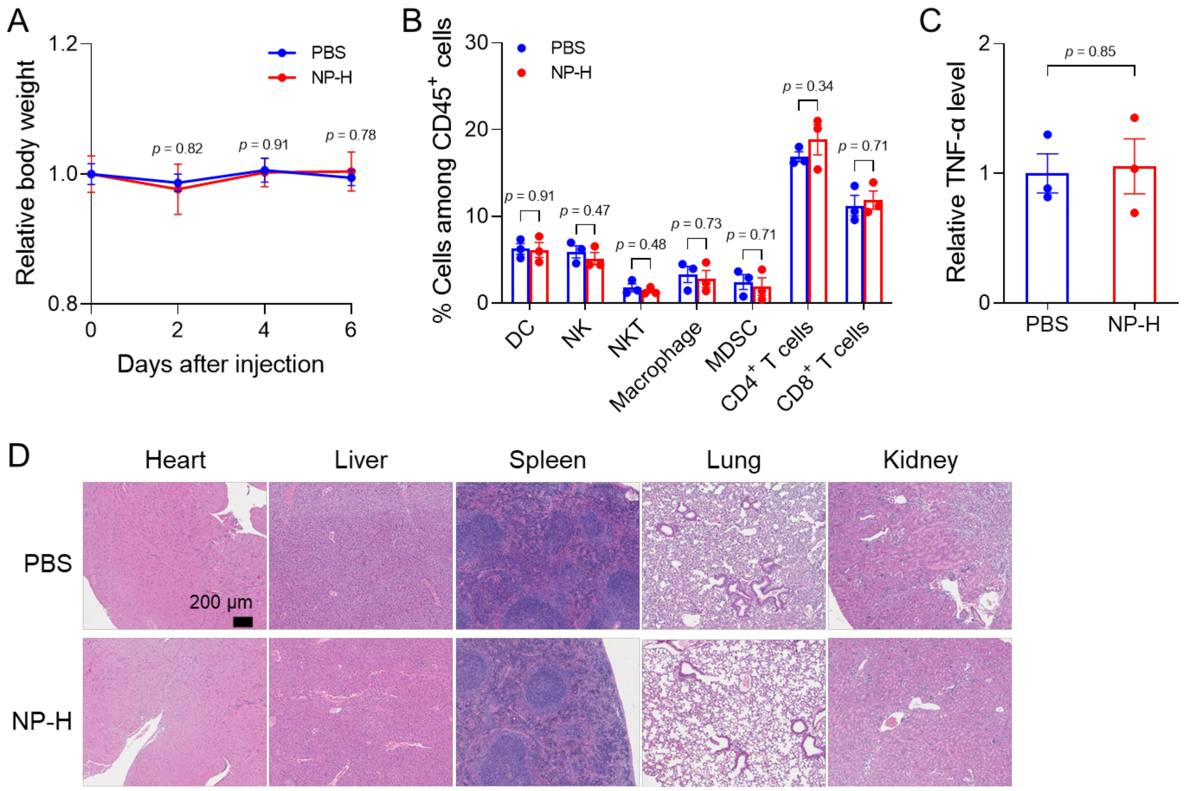

Figure S16. Safety evaluation of NP-H in mice. (**A**) Body weight of Balb/c mice treated with NP-H or PBS. (**B**) Flow cytometric quantification of the percentages of cell subsets (flow cytometric quantification of the number of PBMC cells (DC: $CD11c^+$; NK: $NK\text{-}1.1^+$; NKT: $NK\text{-}1.1^+CD3^+$; macrophage: $CD11b^+F4/80^+$; MDSC: $CD11b^+Gr\text{-}1^+$; $CD4^+$ T cells: $CD3^+CD4^+$; $CD8^+$ T cells: $CD3^+CD8^+$) 6 days following *s.c.* injection of NP-H or PBS in Balb/c mice. (**C**) Serum TNF-α concentration levels from Balb/c mice 6 days following *s.c.* injection of NP-H or PBS. (**D**) Representative H&E staining images of major organs of Balb/c mice treated with NP-H and control (PBS), indicating that NP-H showed no histologically detectable toxicity to these organs. Organs were isolated 6 days following *s.c.* injection of NP-H or PBS. *n* = 3. Data: mean ± s.e.m. *P* values were determined by two-way ANOVA, Tukey's multiple comparison test.

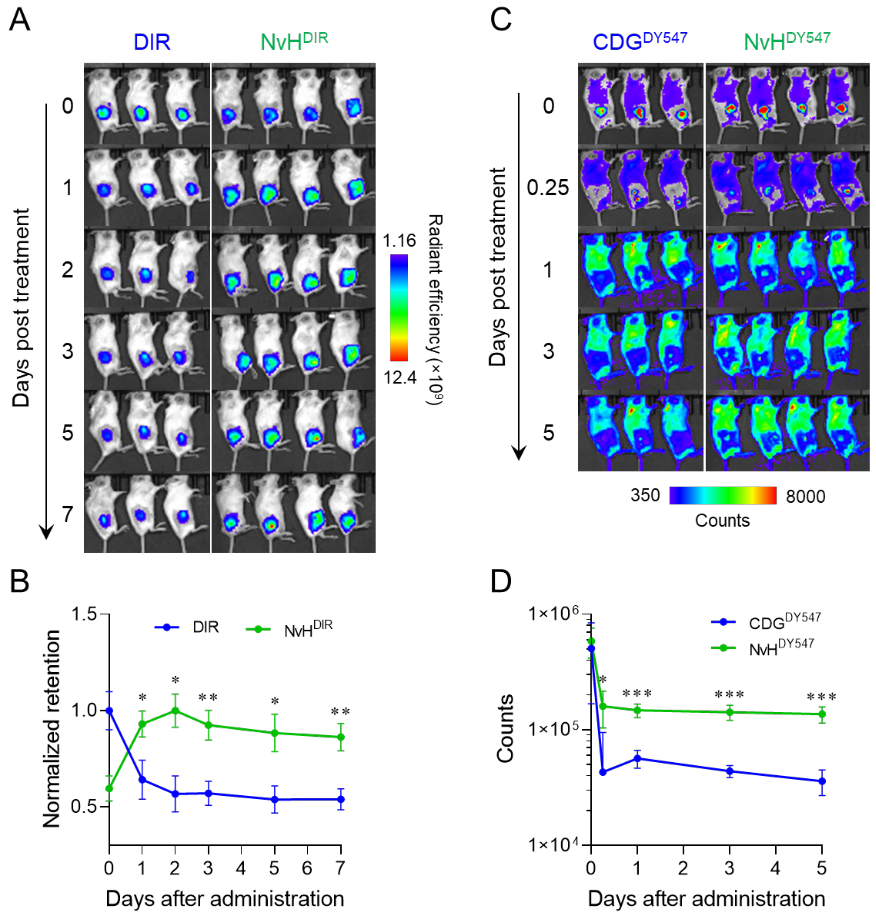

Figure S17. NvH prolonged the tumor retention of immunostimulants. (**A**) IVIS images of 4T1 tumor-bearing Balb/c mice over 1 - 7 days post *i.t.* administration of DIR-loaded NvH (NvH^{DIR}; $n = 4$) or free DIR ($n = 3$) that was used to mimic a small molecular immunostimulant in terms of pharmacokinetics. (**B**) Quantification of DIR fluorescence intensities in the 4T1 tumors in (**A**). (**C**) IVIS images of 4T1 tumor-bearing Balb/c mice over 1 - 5 days post *i.t.* administration of CDG^{DY547}-loaded NvH (NvH^{DY547}; $n = 4$) or free CDG^{DY547} ($n = 3$). (**D**) Quantification of CDG^{DY547} fluorescence intensities in the above 4T1 tumors in (**C**). Data: mean ± s.e.m. *P* values were determined by one-way (**B**) or two-way (**D**) ANOVA, Tukey's multiple comparison test (*$p < 0.05$, **$p < 0.01$, ***$p < 0.001$).

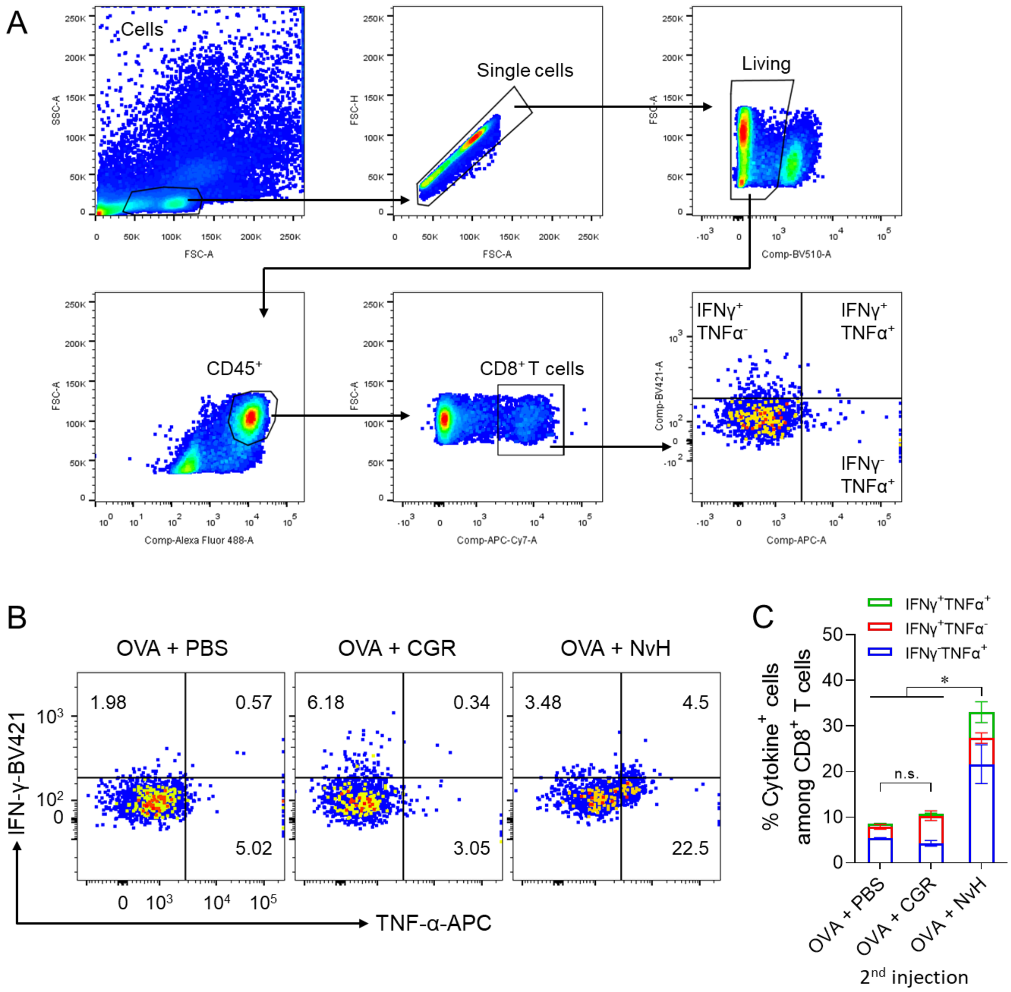

Figure S18. Intracellular cytokine staining in T cells from NvH-immunized mice. (**A**) Representative gating strategy used in cytokine$^+$CD8$^+$ T cell response analysis. (**B**, **C**) Representative flow cytometry plots (**B**) and quantification (**C**) of the cytokine$^+$CD8$^+$ T cell staining results showing that NvH augments cytokine-expressing functional CD8$^+$ T cells in mice (day 4). Cells were restimulated with SIINFEKL peptide/brefeldin A. $n = 3$. Data: mean ± s.e.m. P values were determined by two-way ANOVA, Tukey's multiple comparison test (*n.s.*: not significant; $*p < 0.05$).

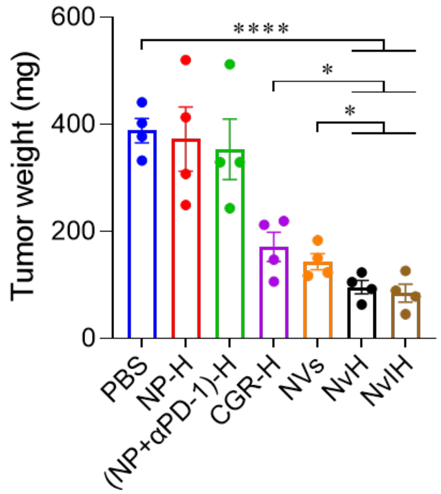

Figure S19. 4T1 tumor mass after 4 days of treatment as shown in Figure 4. ICB in NvIH was αPD-1. Data: mean ± s.e.m. *P* values were determined by one-way ANOVA, Tukey's multiple comparison test (**p* < 0.05; *****p* < 0.0001).

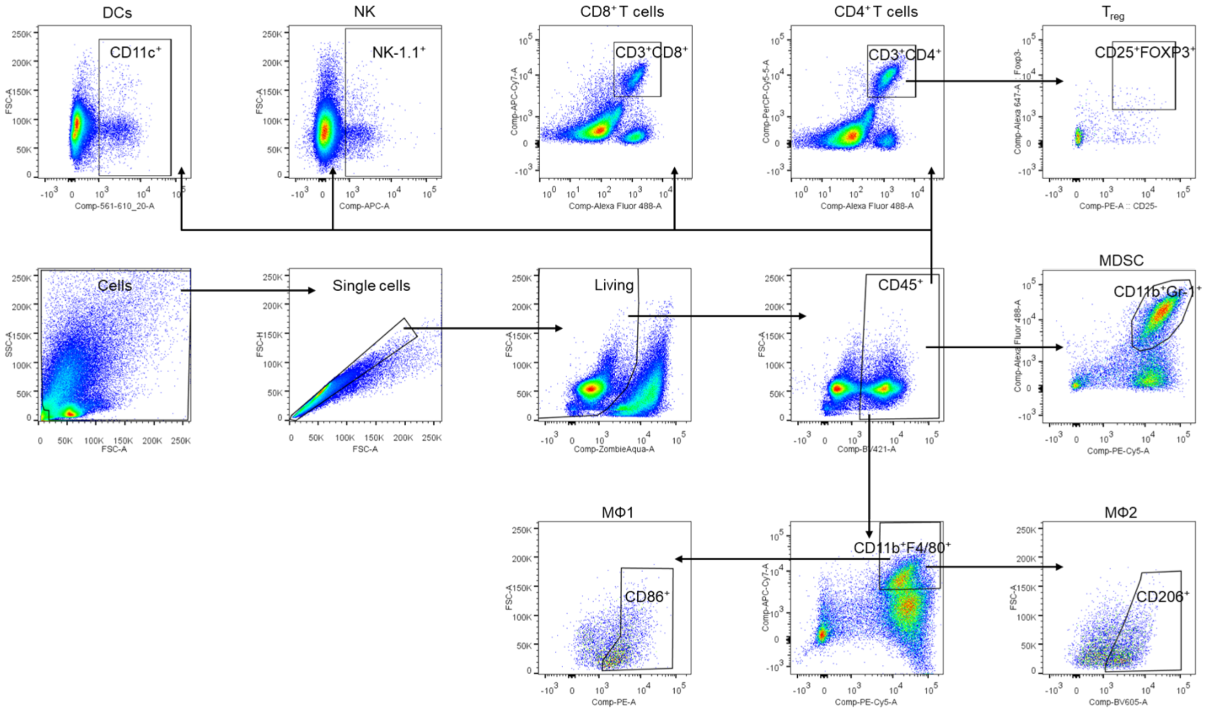

Figure S20. Representative gating tree used in immune analysis.

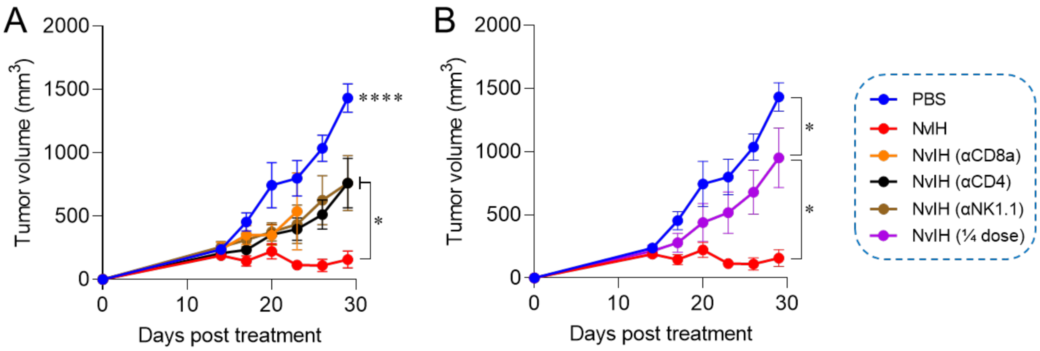

Figure S21. Impact of lymphocyte depletion and doses on 4T1 tumor therapeutic efficacy of NvIH in syngeneic mice. (**A**) Antibody-mediated depletion of NK cells, CD4$^+$ cells, and CD8a$^+$ cells in mice reduced the 4T1 tumor therapeutic efficacy of NvIH in syngeneic mice. Therapeutic treatment started when the average tumor sizes were ~200 mm^3. Depletion antibodies were *i.p.* injected 4 days before treatment and every 3 days thereafter and for a total of 5 times (n = 5). (**B**) Reducing the doses of NvIH reduced its tumor therapeutic efficacy. ICB agents in NvIH were αPD-1 and αCTLA-4. Data: mean ± s.e.m. P values were determined by one-way (**B**) or two-way (**A**) ANOVA, Tukey's multiple comparison test (*p < 0.05; ****p < 0.0001 between PBS and NvIH).

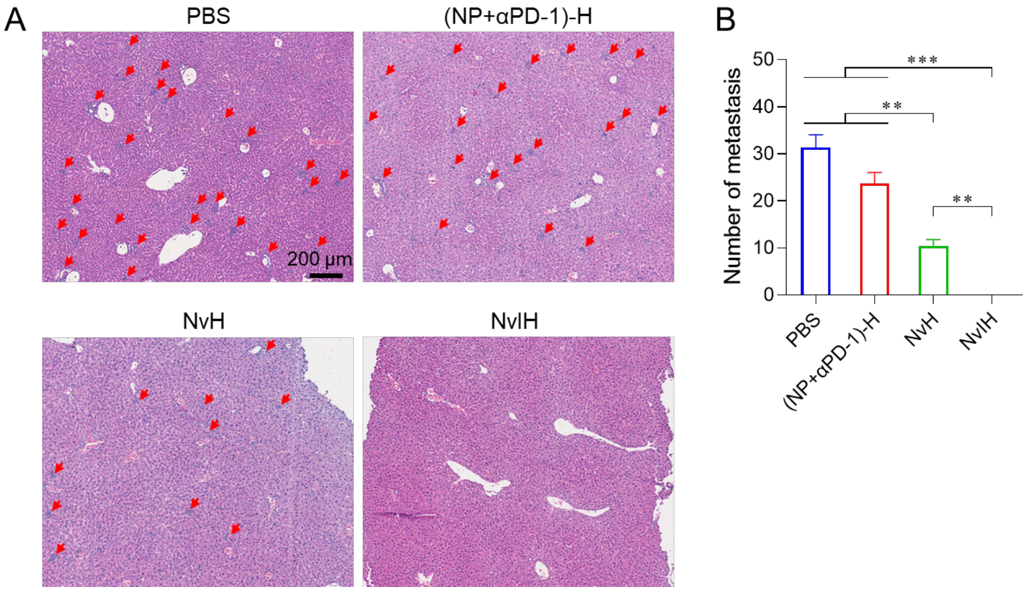

Figure S22. Liver metastasis of 4T1-tumor-bearing Balb/c mice treated with NvIH and controls. (**A**) H&E staining images of livers (red arrows denote metastasis). (**B**) Quantification of tumor metastasis in the livers in (**A**). Three different areas in each H&E image were chose for quantification. These results suggest that NvIH inhibited the liver metastasis of 4T1 cancer cells. ICB in NvIH was αPD-1. Data: mean ± s.e.m. P values were determined by two-way ANOVA, Tukey's multiple comparison test (**p < 0.01; ***p < 0.001).

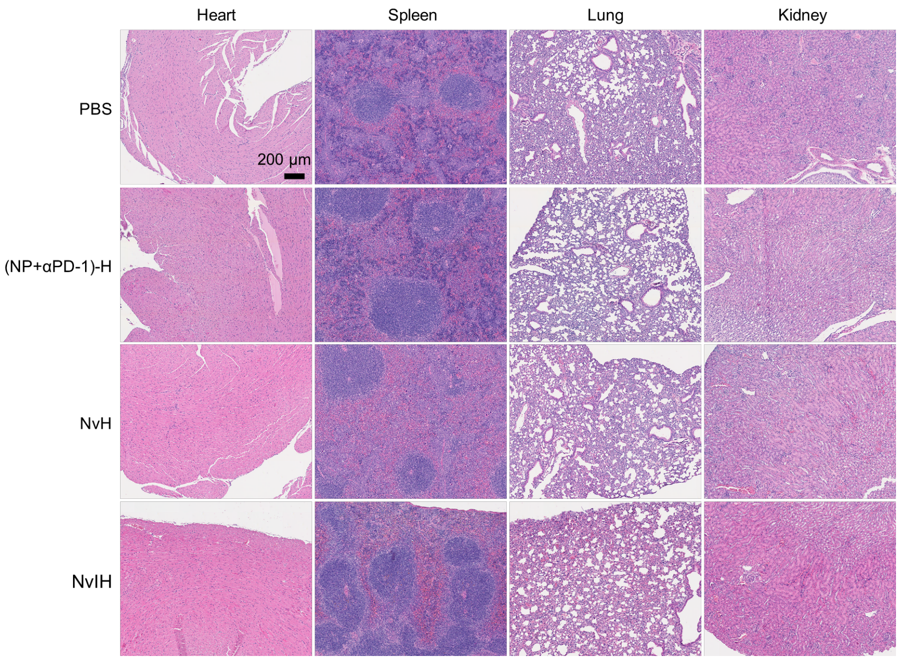

Figure S23. H&E staining images of major organs of 4T1-tumor-bearing Balb/c mice treated with NvIH and controls. Results indicated that NvH or NvIH caused no histologically detectable toxicity to these organs. ICB in NvIH was αPD-1.

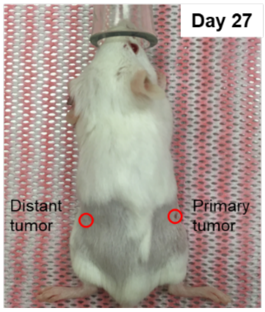

Figure S24. Representative photograph of 4T1-bearing Blab/c mice treated with NvIH. ICB agent in NvIH was αPD-1.

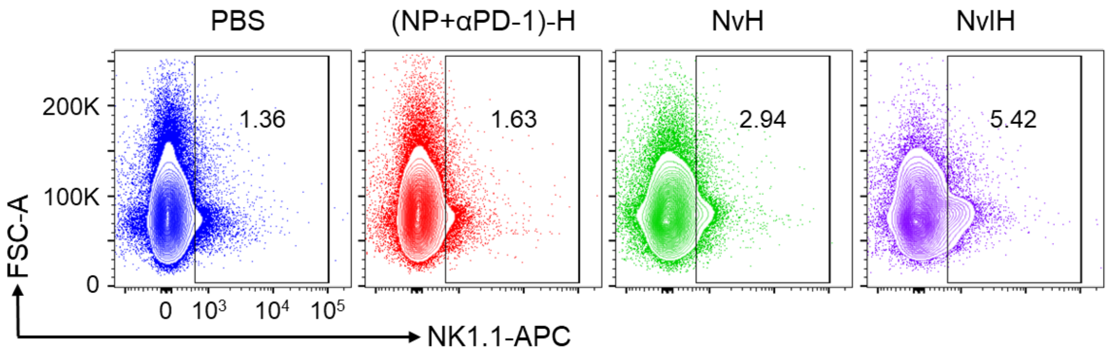

Figure S25. **Representative flow cytometry dot plots of NK cells among CD45$^+$ cells.** ICB agent in NvIH was αPD-1.

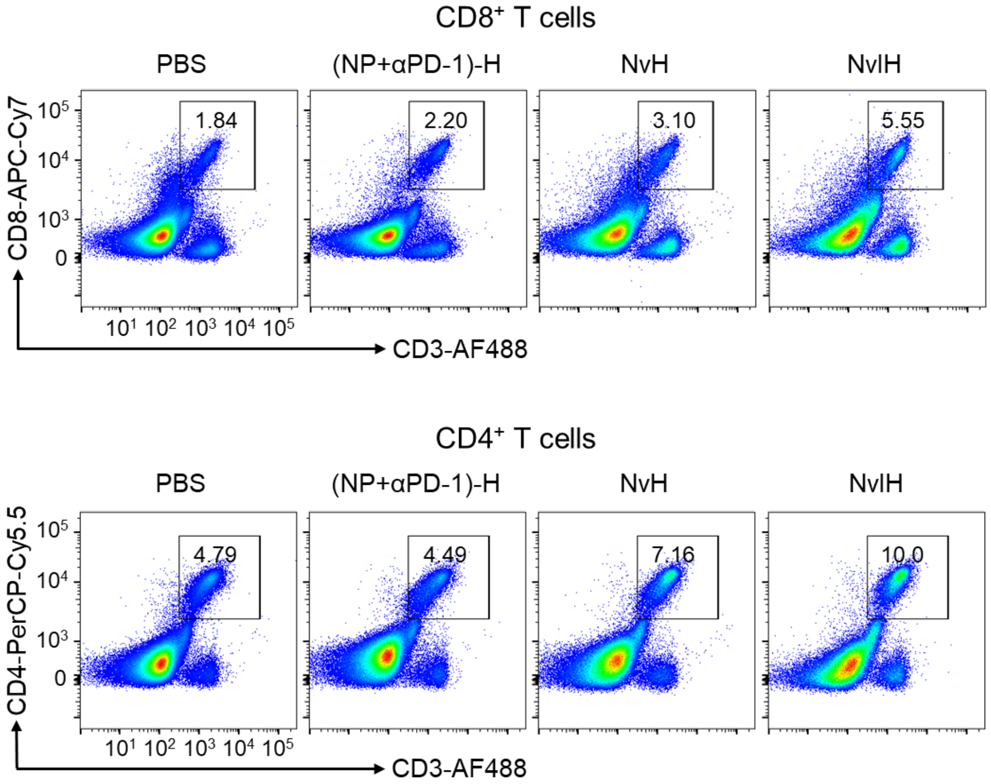

Figure S26. **Representative flow cytometry dot plots of CD3$^+$CD8$^+$ T cells and CD3$^+$CD4$^+$ T cells among CD45$^+$ cells.** ICB agent in NvIH was αPD-1.

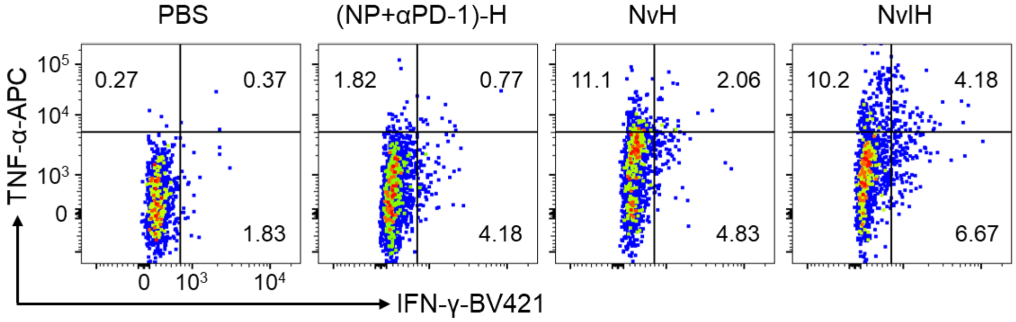

Figure S27. **Representative flow cytometry dot plots of cytokine$^+$CD8$^+$ T cells among all CD8$^+$ T cells.** ICB agent in NvIH was αPD-1.

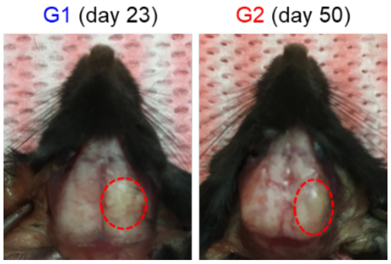

Figure S28. **Representative photographs of GBM tumors treated with PBS (G1) or single-dose lipo-CRG + αPD-L1 + αCTLA-4 (G2).** The days indicate the number of days post tumor cell inoculation.

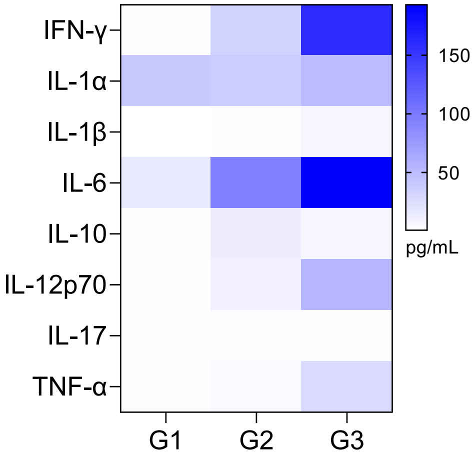

Figure S29. Serum cytokine and chemokine levels measured by Luminex 4 days post tumor treatment in mice. GL261-GFP-Luc GBM tumors-bearing C57BL/6 mice were intracranially treated with PBS (G1), single-dose lipo-CRG + αPD-L1 + αCTLA-4 (G2) or NvIH (G3). ICB agents in NvIH were αPD-L1 and αCTLA-4. *n* = 5.

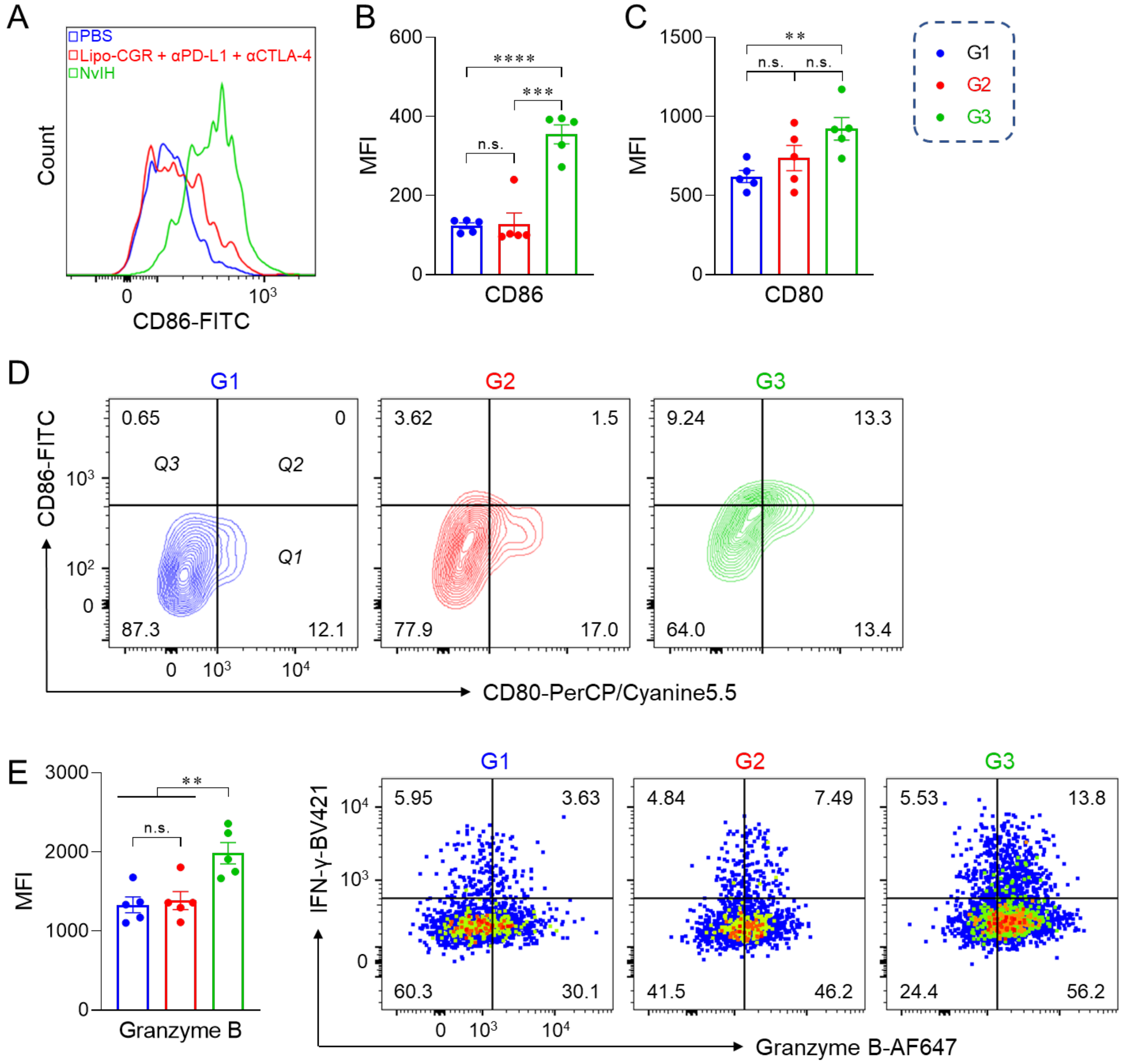

Figure S30. PBMC antitumor immune analysis in NvIH-treated GL261-GFP-Luc GBM-bearing mice. (**A**) Representative flow cytometry histogram of CD86 on DCs from PBMCs. (**B**, **C**) MFI of costimulatory factors CD86 (**B**) and CD80 (**C**) on DCs from PBMCs. (**D**) Representative flow cytometry dot plots of CD80 and CD86 on DCs from PBMCs. (**E**) MFI of granzyme B (left) and representative flow cytometry dot plots of granzyme B and IFN-γ (right) in $CD8^+$ T cells from PBMCs. G1: PBS; G2: lipo-CGR + αPD-L1 + αCTLA-4; G3: NvIH. ICB agents in NvIH were αPD-L1 and αCTLA-4. Data: mean ± s.e.m. *P* values were determined by two-way ANOVA, Tukey's multiple comparison test (*n.s.*: not significant; $^{**}p < 0.01$; $^{***}p < 0.001$; $^{****}p < 0.0001$).

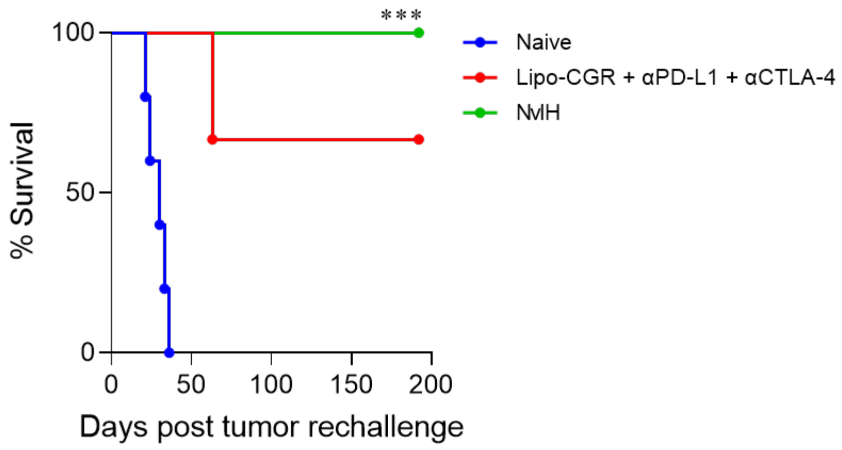

Figure S31. **Survivals of rechallenged dual GL261-GFP-Luc GBM tumors-bearing C57BL/6 mice with complete tumor regression after NvIH immunotherapy.** These mice were rechallenged with GL261-GFP-Luc cells 74 days after the first tumor inoculation. Naive mice (n = 5) were age-matched. Lipo-CGR + αPD-L1 + αCTLA-4: n = 3; NvIH: n = 6. ICB agents in NvIH were αPD-L1 and αCTLA-4. Log-rank (Mantel-Cox) test was used for animal survival comparison (***$p < 0.001$ between naive and NvIH).

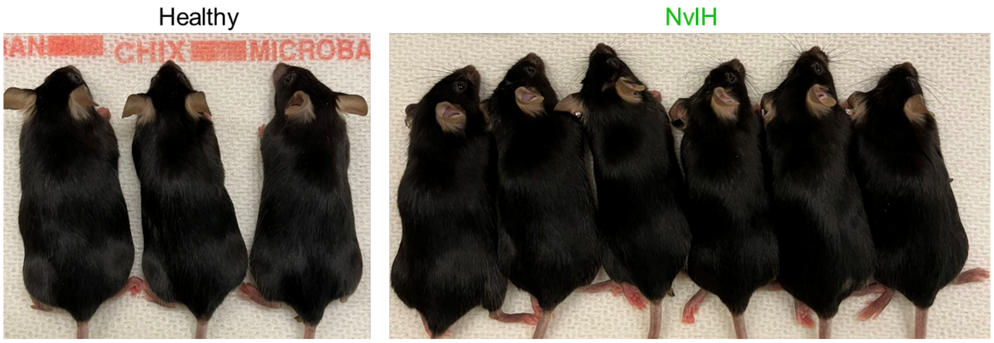

Figure S32. **Photographs of healthy mice and GL261-GFP-Luc GBM-rechallenged C57BL/6 mice.** Left: healthy C57BL/6 mice. Right: dual GL261-GFP-Luc GBM tumors were cured by NvIH in C57BL/6 mice, and then rechallenged with GL261-GFP-Luc tumor cells; shown in the photograph are the above mice 135 days after GL261-GFP-Luc rechallenge. ICB agents in NvIH were αPD-L1 and αCTLA-4.
